# Development and psychometric validation of a novel scale for measuring ‘psychedelic preparedness’

**DOI:** 10.1038/s41598-024-53829-z

**Published:** 2024-02-08

**Authors:** Rosalind G. McAlpine, George Blackburne, Sunjeev K. Kamboj

**Affiliations:** 1 Clinical Psychopharmacology Unit, Clinical, Educational and Health Psychology, University College London, London, UK; 2 Experimental Psychology, University College London, London, UK

**Keywords:** Human behaviour, Psychiatric disorders, Risk factors

## Abstract

Preparing participants for psychedelic experiences is crucial for ensuring these experiences are safe and, potentially beneficial. However, there is currently no validated measure to assess the extent to which participants are well-prepared for such experiences. Our study aimed to address this gap by developing, validating, and testing the Psychedelic Preparedness Scale (PPS). Using a novel iterative Delphi-focus group methodology (‘DelFo’), followed by qualitative pre-test interviews, we incorporated the perspectives of expert clinicians/researchers and of psychedelic users to generate items for the scale. Psychometric validation of the PPS was carried out in two large online samples of psychedelic users (N = 516; N = 716), and the scale was also administered to a group of participants before and after a 5–7-day psilocybin retreat (N = 46). Exploratory and confirmatory factor analysis identified four factors from the 20-item PPS: Knowledge-Expectations, Intention-Preparation, Psychophysical-Readiness, and Support-Planning. The PPS demonstrated excellent reliability (ω = 0.954) and evidence supporting convergent, divergent and discriminant validity was also obtained. Significant differences between those scoring high and low (on psychedelic preparedness) *before* the psychedelic experience were found on measures of mental health/wellbeing outcomes assessed *after* the experience, suggesting that the scale has predictive utility. By prospectively measuring modifiable pre-treatment preparatory behaviours and attitudes using the PPS, it may be possible to determine whether a participant has generated the appropriate mental ‘set’ and is therefore likely to benefit from a psychedelic experience, or at least, less likely to be harmed.

## Introduction

Psychedelic drugs have recently re-emerged in scientific and popular culture as potentially powerful empirical tools to probe the properties of the human mind, as well as to treat a myriad of neuropsychiatric conditions^1–3^. Psychedelics initiate perhaps uniquely radical alterations in perception and behaviour, with their complex phenomenology ranging from feelings that one is dying^4^, to vivid reliving of past traumatic events^5^, to interacting with divine or malevolent entities^6^. Accordingly, the need to prepare for upcoming psychedelic experiences is being increasingly acknowledged by researchers and practitioners as essential^7–11^. As such, the typical structure of a modern psychedelic trial will involve an explicitly mentioned ‘preparation phase’ intended to generate appropriate expectations, and to provide participants with strategies to optimise their upcoming psychedelic experience. This phase usually involves psychoeducation, the exploration of intentions and motivations, and the cultivation of a trusting therapeutic alliance^12–15^. These diverse and often non-standardised preparatory session components^16^ are thought to be important determinants of treatment efficacy.

A common aim of psychedelic preparation is to foster a state preceding the psychedelic session that is conducive to a safe and personally meaningful experience; a state we refer to here as *psychedelic preparedness*. Individuals in this state feel psychologically, physically, and socially ‘ready’ for both the content and consequences of the experience. Despite a growing need for standardised measures of psychedelic preparedness, researchers have paid little attention to the methods by which such “extensive psychological preparation”^17^ (pp. 726) is evaluated, and no such measure has been developed for clinical research or practice. More generally, the development of measurement scales in psychedelic science – and indeed, psychological science more broadly – has tended to be driven by expert consensus, or the even narrower perspectives and intuitions of the scale developers. However, there is an increasing recognition that a failure to consider the perspective of those with first-hand experience leads to an impoverished understanding of the phenomenon of interest^18–20^.

The present article describes the development of a novel, freely available, self-rated measure of psychedelic preparedness – The Psychedelic Preparedness Scale (PPS). This is the first scale that we are aware of whose items focus primarily on the pre-psychedelic period rather than the acute experience or psychological changes occurring after the experience. In designing this scale, we aimed to ensure that initial item selection was guided by a wide set of expert opinions, and therefore devised a novel iterative strategy involving using both a Delphi methodology with learned experience experts and focus groups with lived experience experts. We additionally employed the recently developed ‘pre-test interview’ approach^21,22^, which aims to develop detailed (qualitative) insights into participants’ (end users') experiences of survey questions, and to refine these in response to the insights gained during the interview.

Given the proposed critical (though as yet, unproven) role of preparedness in enabling participants to navigate and shape a psychedelic episode into a potentially transformative experience, a demonstration of sound psychometric properties of the PPS could facilitate future systematic investigations into the role of psychedelic preparedness in predicting acute and longer-term responses to psychedelics.

## General methods

All research was performed in accordance with the Declaration of Helsinki and all procedures were reviewed by, and received approval from, the University College London Research Ethics Committee (9437/002). All participants provided electronic informed consent at the beginning of each study. In this process, participants were presented with a clear outline of the study's purpose, procedures, their rights, and any associated risks or benefits. Consent was obtained by participants checking boxes to acknowledge their understanding and agreement before proceeding with the survey. All studies were performed in line with relevant guidelines and regulations^23–25^.

Following best practice recommendations for scale development and validation^23–25^, we provide a detailed methodological account of the item generation and scale development process (Study 1), as well as results from a psychometric validation study employing two distinct online datasets (Study 2), and a preliminary implementation of the PPS with a sample of psilocybin retreat participants (Study 3). In the section below we provide methodological details relevant to all three studies. Additional methodological details relevant to the specific studies are then provided under separate headings. All data was collected online.

### Participants

Participants in all three studies were required to meet the inclusion criteria of being over 18 years of age and having a proficient understanding of the English language. Recruitment was carried out through online methods, specifically by posting targeted advertisements on social media platforms. Detailed information regarding the inclusion criteria and recruitment process specific to each study can be found under separate headings.

### Data analysis

Descriptive statistics for each study are presented as frequencies (and %). All statistical analyses were conducted using RStudio Version 2022.12.0 + 353^26^.

### Measures

#### Emotional breakthrough

The Emotional Breakthrough Inventory (EBI)^27^ assesses, retrospectively, episodes of catharsis or emotional release following a psychedelic experience. It is a 6-item scale scored on 0 to 100 visual analogue scale (VAS). Here, EBI scores are presented as a single total, summed score ranging from zero to 600.

#### Mystical experience

The Mystical Experience Questionnaire (MEQ)^28^ is a 30-item scale scored on a 6-point Likert scale (1 = none, not at all; 6 = extreme, more than any other time in my life). It consists of four factors which measure different dimensions of the mystical-type experience: ‘mystical’, ‘positive-mood’, ‘transcendence of time and space’ and ‘ineffability’. In the current study, we present data in the form of a single total MEQ score ranging from 30 to 180.

#### Challenging psychedelic experience

The Challenging Experience Questionnaire (CEQ)^29^ is a 26-item scale scored on a 6-point Likert scale (1 = none, not at all; 6 = extreme, more than any other time in my life). It consists of seven factors which measure different dimensions of challenging aspects of the psychedelic experience: ‘fear’, ‘grief’, ‘physical distress’, ‘insanity’, ‘isolation’, ‘death’, ‘paranoia’. Here, we present data in the form of a single total CEQ score ranging from 26 to 156.

#### Post-psychedelic growth

The Post-Traumatic Growth Inventory Short Form (PTGI-SF)^30^ is a 7-item scale that assesses positive self-related changes (‘growth’) experienced by individuals following a traumatic event. While the scale was developed and has been used to assess growth following traumatic events, a review of the items of the PTGI suggest that it also has broader relevance, including for participants who have experienced positively valenced transformative events. We therefore minimally adapted it for use in studying the effects of psychedelic experiences (see Supplementary Material A1.1). Despite the items being identical to the PTGI-SF, to distinguish its use in the context of psychedelic growth from its common use to assess posttraumatic growth, we refer to the scale here as the Post-Psychedelic Growth Inventory (PPGI). Respondents rate their beliefs on a 7-point Likert scale (1 = strongly disagree; 7 = strongly agree), with items including changes in priorities, spiritual matters, closeness with others, and personal strength (e.g. “I have changed my priorities about what is important in life”, “I have a better understanding of spiritual matters”, “I have a greater sense of closeness with others”, “I discovered that I'm stronger than I thought I was” etc.,). Here, we present data in the form of a single total PPGI score ranging from 7 to 49.

#### Centrality of event

The 10-item Centrality of Event Scale (COES)^31^ is a validated measure of how much a specific experience becomes central to personal identity, a turning point in one's life story, and a reference point for everyday inferences. Respondents rate statements such as "The experience permanently changed my life" and "The experience was a turning point in my life" on a 7-point Likert scale (1 = strongly disagree; 7 = strongly agree). Although traditionally used to assess growth following traumatic events, it was minimally adapted for use in studying the effects of psychedelic experiences, in line with the PPGI justification above (see Supplementary Material A1.2). Here, we present data in the form of a single total COES score ranging from 7 to 70.

#### Wellbeing

The Short Warwick-Edinburgh Mental Wellbeing Scale (SWEMWBS)^32^ is a 7-item measure of wellbeing. For this study, the instructions of the SWEMWBS were slightly adapted to specifically capture data related to changes in wellbeing following psychedelic experiences. Participants were asked to select the answer that best represented how they had felt since their most significant psychedelic experience, and the items were modified to include indications of change (e.g. "I've been feeling more optimistic about the future", "I've been feeling more useful", "I've been feeling more relaxed", etc.). Each item was rated on a 7-point Likert scale (1 = definitely disagree; 7 = definitely agree), and a total SWEMWBS score was calculated for each participant, ranging from 7 to 49.

#### Romantic attachment style

The Experiences in Close Relationship Scale-Short form (ECR-S)^33^ is a 12-item scale scored on a 7-point likert scale (1 = strongly disagree; 7 = strongly agree), which provides data on two continuous scales concerning the extent to which participants show attachment dimensions: anxiety and avoidance. For the purposes of this paper, the instructions of the ECR-S were slightly adapted to specifically capture data related to changes in romantic attachment style following psychedelic experiences, by asking participants how they experienced romantic relationships *before* the stated psychedelic experience. As only the scores related to the anxiety attachment dimension were included (e.g. “I needed a lot of reassurance that I am loved by my partner”, “I find that my partner(s) don't want to get as close as I would like”, “My desire to be very close sometimes scares people away” etc.,), we refer to this scale as the ECR-SAn. A total ECR-SAn score was calculated for each participant, ranging from 7 to 42.

#### Extraversion

The Big Five Inventory (BFI)^34^ is a 44-item scale scored on a 5-point likert scale (1 = strongly disagree; 5 = strongly agree), which measures an individual on the Big Five Factors (dimensions) of personality^35^. As only the 8 items related to Extraversion were included (e.g. “Is talkative”, “Is full of energy”, “Generates a lot of enthusiasm” etc.,), we refer to this scale as the BFI-Ex. A total BFI-Ex score was calculated for each participant, ranging from 8 to 40.

#### Altered states of consciousness

The Altered States of Consciousness (11D-ASC)^36^ was used to assesses the acute effects of psilocybin experiences on 11 dimensions of consciousness, including ‘experience of unity’, ‘spiritual experience’, ‘blissful state’, ‘insightfulness’, ‘disembodiment’, ‘impaired control and cognition’, ‘anxiety’, ‘complex imagery’, ‘elementary imagery’, ‘audio-visual synaesthesia’, and ‘changed meaning of percepts’. Items contain statements (e.g. “I felt I was in a wonderful other world”), which can be answered on a continuous scale ranging from “No, not more than usual” (0) to “Yes, much more than usual” (100), where 0 is considered to resemble a sober state. Separate mean subscale scores were calculated, ranging from 0 to 100. The mean score of all the questions gives the global-ASC score.

#### Depression, anxiety, and stress

The Depression, Anxiety, and Stress Scale (DASS-21)^37^ was used to assess symptoms of depression, anxiety, and stress. The DASS-21 consists of 21 items rated on a 4-point Likert scale (1 = did not apply to me at all, 4 = applied to me very much, or most of the time). Separate total scores were calculated for each subscale. The total score of each subscale can range from zero to 21.

## Study 1: item generation and scale development

### Methods

#### Preliminary consultation phase

Prior to the Delphi-focus group (DelFo) and qualitative pre-test interviews (QPIs), six professionals with expertise in psychedelics from clinical psychiatry, philosophy, and psychedelic research were consulted to generate an initial item pool for the scale. Information about participants’ characteristics were not elicited for this preliminary phase. These consultations included online feedback sessions to gather views on the definition of psychedelic preparedness, its subdomains, and items that should be considered for inclusion in the scale. Based on feedback from these preliminary consultations, the research team generated 56 items covering 10 sub-domains of preparatory behaviours and attitudes through rapid thematic analysis^38,39^ (Education, Intention Setting, Expectation Management, Psychological Mindedness, Emotional Readiness, Willingness to Surrender, Psychophysical Robustness, Safety/Security, Prepared for Change, Preparatory Practises) and three separate items covering preparatory efficacy. These 59 items were used in the subsequent DelFo and QPIs for methodological refinement of the scale. Each phase had a unique set of participants with no overlap.

### Participants

#### Delphi-focus group (DelFo) method

‘Expert judges’ (N = 7; researchers and clinicians with expertise in the psychedelic domain) for the Delphi rounds were recruited via the authors’ professional network/word of mouth. They were required to be actively involved in psychedelic research or psychedelic-assisted psychotherapy practice. Demographic and professional characteristics of expert judges are presented in the supplement (Supplementary Material A2.1). None of these judges were involved in the preliminary consultations.

‘Lived experience judges’ (N = 8; individuals with experience of psychedelic assisted psychotherapy (PAP)) for the focus groups were recruited via advertisements displayed on social media platforms. Participants were invited to attend all three focus group rounds. Demographic characteristics and PAP experience of lived experience judges are presented in the supplement (Supplementary Material A2.2).

#### Qualitative pre-test interviews (QPIs)

An additional purposive sample of six lived experience participants were recruited for the QPIs through collaborating psilocybin retreat centres (The Buena Vida (Mexico) [N = 3]], Earth Awareness (Netherlands) [N = 3]). All participants were required to have previously attended a 5–7-day psilocybin retreat. Demographic characteristics and PAP experience of these participants are presented in the supplement (Supplementary Material A2.3).

### Procedures

#### DelFo method

**Figure 1:**
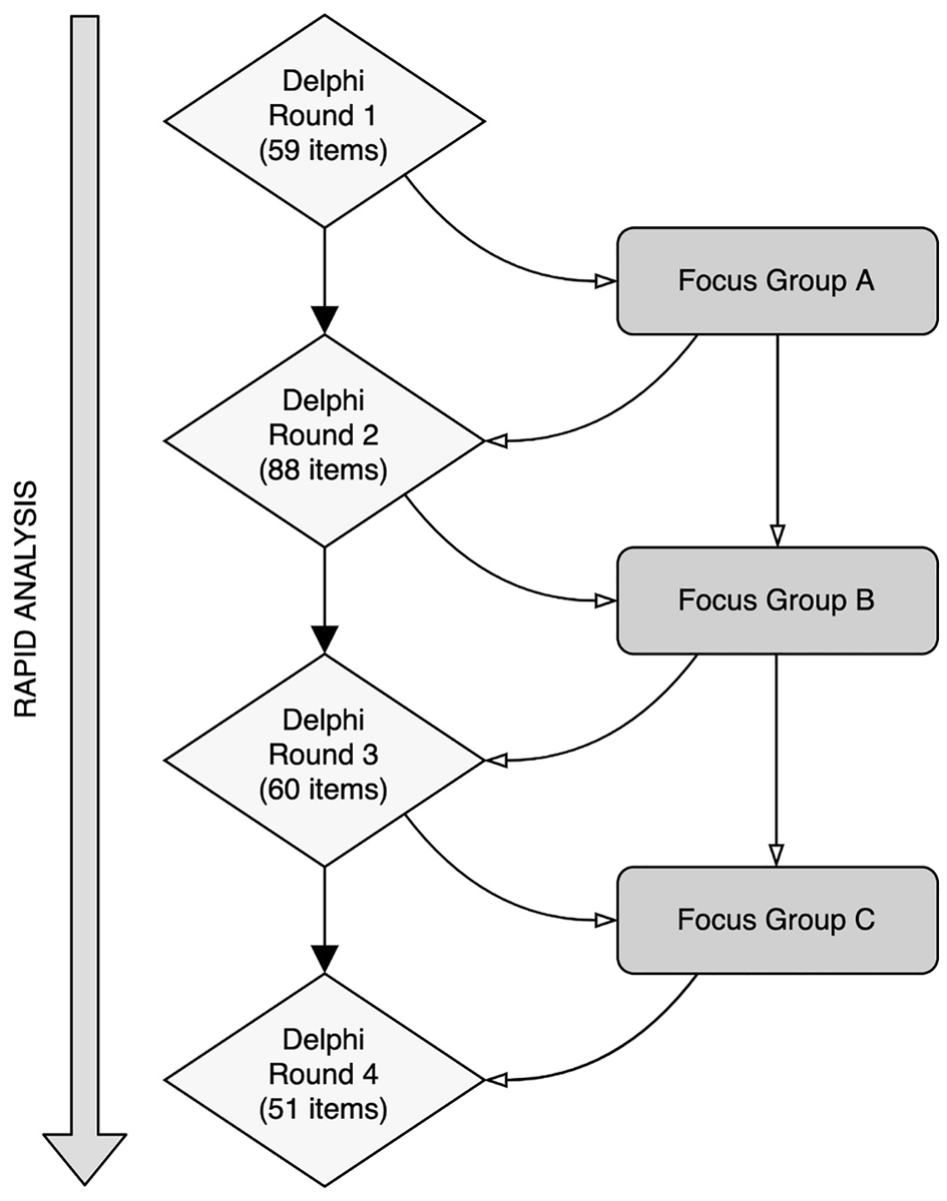
The ‘DelFo Method’. Flow diagram illustrating our integration of Delphi Method with focus group discussions. The number of items evaluated in each iterative Delphi round is indicated in the diamonds.

We developed a novel Delphi-focus group (‘DelFo’) method to evaluate each of the initial items developed during the preliminary consultation phase for relevance, representativeness, and technical quality (e.g. lack of ambiguity, comprehensibility, use of inclusive language etc.,). This involved iterative and interactive evaluation by both expert judges, who participated in multiple Delphi rounds, and lived experience judges, who participated in multiple interspersed focus group discussions (Fig. 1). To complete the Delphi process, participants were required to respond across four iterative rounds of consultation^40,41^. We judged this approach to be the optimal method for encouraging an enriching and responsive exchange between those with lived and learned experience. The approach of convening the two groups of participants in separate interspersed focus groups (lived experience judges) between Delphi rounds (expert judges) was intended to ensure that potential perceived differences in status, knowledge, and power, did not impede a creative flow of ideas. The use of multiple rounds allowed responses from earlier (Delphi) rounds to be incorporated as new items for consideration in subsequent rounds and re-evaluated in light of discussion in Round 4. Our decision to specifically use four Delphi rounds and three interspersed lived experience focus groups was informed by practical considerations, as well as the likelihood that this would be sufficient to arrive at a consensus in item selection.

In Round 1 of the Delphi procedure, the ‘expert judges’ independently rated the extent to which they agreed that each of the 59 items developed during the preliminary consultation phase should be included in the final scale. Each item was rated using a 4-point Likert scale (1 = strongly agree; 4 = strongly disagree) A free-text response was available to participants within each of the survey sub-domains, providing the opportunity to elaborate or explain responses (e.g. to suggest wording changes or strongly advocating for or against the inclusion of an item). In subsequent rounds (2–4) participants were provided with individualised surveys which included their responses from the preceding round, and the group’s responses (% agreeing/disagreeing to item inclusion). In rounds 2 to 4, items were added or removed, reflecting the evolving consensus from the feedback of earlier rounds. This process, inherently responsive to the expert opinions, led to a final decision to include 51 items for further psychometric evaluation. This number was not predetermined but emergent from the iterative nature of the Delphi method.

After each Delphi round, the expert judges’ responses were fed back to the lived experience judges during focus groups discussions (with author RM) who (re)considered their views regarding the scale content in light of the expert judges’ responses^42^. A focus group methodology was chosen due to its utility in eliciting views from relatively rare participant groups (e.g. recipients of psychedelic therapy), provision of a safe discussion forum regarding difficult and potentially stigmatised topics (e.g. use of psychedelic substances), capacity for problem solving (e.g. adapting questionnaire items based on accessibility and representativeness) and the generation of new ideas (e.g. generating new items to be included within novel questionnaires)^43^. Each focus group discussion lasted between 60 and 90 min and took place via teleconferencing (Zoom) and used an auto-transcription feature to capture the contents of the discussion (audio was also recorded for content verification purposes). Sessions were guided by an indicative schedule with a limited number of guiding questions based on the results of the previous Delphi round. Feedback derived from the focus group discussions was incorporated into each round of the Delphi study.

#### Qualitative pre-test interviews (QPIs)

The results of the DelFo method fed into the a qualitative pre-test interview (QPI) approach^22^ which was used to assess the extent to which (a) the questions reflected the domain of interest (psychedelic preparedness) and (b) answers produced valid measurements^44^. The QPI is a relatively new method that involves assigning "co-expert" responsibility to interviewees and using a "think-aloud" approach, where participants share their thoughts on each item as they read through them. This collaborative process between the interviewer and the participant aims to identify difficulties or vagueness in the survey items for refinement and improvement of the measure.

We administered draft survey questions to six new participants with previous experience of psychedelic treatment (Supplementary Material A2.3). During the QPI, they were asked to verbalise the mental process entailed in providing answers. QPIs were conducted with each participant individually via teleconferencing, with similar recording and transcription as described above.

During the QPI, items from the draft survey questions were carefully reviewed and modified as needed to ensure that participants understood the questions as intended and were able to provide answers that reflected their experience. This involved making changes to grammar, word order, word choice, and other aspects of the survey items. For example, if participants expressed confusion about a particular question, the grammar was clarified, or the wording was adjusted to be more concise. Additionally, if participants found certain items difficult to answer, the wording was refined to better capture their experiences. This iterative process continued until saturation was reached, ensuring that the survey items were refined and improved to enhance their validity and reliability.

#### Data analysis

Given the relatively small group of experts used in the Delphi procedure, we took the pragmatic decision to define "consensus" as the majority of participants agreeing/strongly agreeing or disagreeing/strongly disagreeing with a statement (i.e. inclusion of an item)^42,45^. After each round the number of items in each sub-domain and number of items where consensus was achieved (%) was calculated.

Qualitative analysis of the focus groups and QPIs was inspired by a rapid thematic analysis, whereby data collection and preliminary data analysis occurs in tandem^38,39^. This process informed the direction of subsequent focus groups/QPIs and refined the interview guides to capture new information.

### Results

#### DelFo

Table 1 shows the summary of how the number of items in each of the subdomains changed over the course of the rounds of the DelFo procedure. The number of items where consensus was achieved improved for each domain from Round 1 to Round 4. Overall (across domains) consensus increased from 66.1% of items (39 of 59 items) to 92.2% (47 of 51 items) by Round 4. As can be seen in Table 1, 100% consensus was achieved for 7 domains and the lowest level of consensus was 66.7% for Intention Setting.

**Table 1:** Summary of included items by sub-domain over the four rounds of DelFo.

Sub-domain	Number of items in each sub-domain				Number of items where consensus was achieved (%)			
	*R1*	*R2*	*R3*	*R4*	*R1*	*R2*	*R3*	*R4*
Education	4	5	6	5	3 (75%)	4 (80%)	5 (83.3%)	4 (80%)
Intention setting	6	9	5	3	5 (83.3%)	6 (66.7%)	4 (80%)	2 (66.7%)
Expectation management	6	8	4	4	5 (83.3%)	7 (87.5%)	4 (100%)	4 (100%)
Psychological mindedness	0	6	3	3	0 (0.0%)	5 (83.3%)	3 (100%)	3 (100%)
Emotional readiness	8	9	5	4	5 (62.5%)	8 (88.9%)	4 (80%)	3 (75%)
Willingness to surrender	6	8	3	3	4 (66.7%)	7 (87.5%)	3 (100%)	3 (100%)
Psychophysical robustness	5	7	4	3	3 (60%)	6 (85.6%)	3 (75%)	3 (100%)
Safety/security	5	7	4	4	4 (80%)	7 (100%)	4 (100%)	4 (100%)
Prepared for change	6	8	7	6	3 (50%)	8 (100%)	6 (85.7%)	6 (100%)
Preparatory practises	10	11	9	6	6 (60%)	9 (81.8%)	8 (88.9%)	5 (83.3%)
Preparation efficacy	3	10	10	10	1 (33.3%)	10 (100%)	10 (100%)	10 (100%)
Totals	59	88	60	51	39 (66.1%)	77 (87.5%)	54 (90%)	47 (92.2%)

*RX* Round.

#### QPIs

Based on the feedback from QPIs (Supplementary Material 3.1), additional modifications were made to grammar and word choice for four items.

### Conclusion

Upon completion of the DelFo and QPI procedures, a total of 51 items were selected, as detailed in Supplementary Material A3.2. To validate the appropriateness of the preparedness items for both prospective and retrospective versions of the scale, a rigorous psychometric analysis was conducted in Study 2. The ten items related to 'preparedness efficacy' (e.g. "My preparation impacted my experience"), which are applicable only in a retrospective context, were excluded from the analysis and treated as an independent measure of ‘preparation efficacy’.

## Study 2: psychometric validation

### Methods

#### Recruitment

Two large, self-selected groups of participants with experience of psychedelic use took part in validation of the preparedness items generated in Study 1. Data from Sample A (N = 518) was used for the initial extraction of factors (EFA). A proportion of participants from Sample A repeated the survey after 2 weeks (N = 296). These follow-up responses were only used to assess the test–retest reliability of the PPS and were not entered into the factor analyses. Data from Sample B (N = 718) was used for the tests of dimensionality (confirmatory factor analysis; CFA). A pooled sample of 1236 participants were analysed for subsequent tests of reliability and validity.

#### Procedures

Participants responding to the survey link were guided to an online information sheet about the study. After providing informed consent, they completed a structured online survey consisting of the 41 psychedelic preparedness items developed in Study 1, as well as the MEQ, CEQ, EBI, PPGI, COES, SWEBWBS.

The instructions provided to participants for responding to the 41 items were: “In relation to your most significant psychedelic experience, indicate the degree to which you agree with the following statements.” (See Supplementary Material B1.2 for retrospective PPS). Participants were asked o respond on a 7-point Likert scale ranging from “not at all” to “completely” to each item. The same instructions were used for the 10 preparedness efficacy items (Supplementary Material A3.2). Importantly, the survey did not specifically ask participants to identify the substance involved in their most significant psychedelic experience. The rationale behind this decision was that the primary focus of this initial study was on the broader aspects of psychedelic preparedness across various experiences, rather than on the effects of specific substances. This approach allowed us to explore and understand preparedness in a more general context, applicable to a wide range of psychedelic experiences. However, we acknowledge the possibility that differences in psychedelic drug effects may have distinct implications for preparation. We do not explore this in the current study.

After giving informed consent, participants in re-test conditions received email reminders including links to the survey at the second time point. All data obtained were anonymous, and no personally identifying information was collected apart from email addresses – which we required for participants to be sent the second survey link. No data that could identify individuals or their responses have been shared, and no IP addresses were collected.

#### Data analysis

##### Extraction of factors

The 41-item scale was subjected to exploratory factor analysis (EFA) methodology to determine the optimal number of items to be retained and number of factors that fitted the set of items. Kaiser–Meyer–Olkin (KMO) measure of sampling adequacy and Bartlett’s test of sphericity were used to test the sample adequacy for EFA. The number and composition of factors was determined through a consideration of common psychometric heuristics, including visual examination of the scree plot^46^, the ‘Kaiser rule’^47^, optimal coordinate, parallel analysis and acceleration factor tests^48^.

Oblique (oblimin) rotation was used in factor extraction, as this rotation method favours interpretability and allows factors to intercorrelate. The subscales were based on the factors extracted and interpreted through investigation of covariance-based factor loading patterns. To maximise clarity and simplicity in interpretation, two guiding criteria were used: (1) each item assigned to a subscale demonstrated a factor loading of ⋟0.3 and (2) maximum cross loading on any other factor should be < 0.2^49,50^^.^

##### Test of dimensionality

Having identified the items to retain (based on (cross)loadings) and a likely factor structure underlying these observed items from the EFA, tests of dimensionality were conducted on a separate dataset (Sample B) to validate the identified factor structure. CFA was conducted on the following structural models: (1) The hypothesised 4-factor model structure, as determined through EFA; (2) A hierarchical 4-factor model, i.e. a version of the 4-factor model in which the covariance between the first-order latent variables was described by an overall second-order latent construct labelled “Psychedelic Preparedness”; (3) A 3-factor model, constructed by observing the highest factor covariance in the hypothesised model; (4) A 2-factor model, constructed by again observing the highest factor covariance in the 3-factor model. Nested models 2–4 were compared to the hypothesised 4-factor model.

Goodness of model fit was based on a consideration of the model chi-square statistic to assess the absolute fit of the model to the data. In addition however, because the chi-square statistic is heavily influenced by sample size, we additionally used the Comparative Fit Index (CFI), Tucker-Lewis Index (TLI), Root Mean Square Error of Approximation (RMSEA) and standardised root mean square residual (SRMR) values to determine goodness of model fit^51,52^. Commonly used criteria (e.g. CFI/TFI values ≥ 0.95 to indicate good fit; ≥ 0.90 to indicate adequate fit) were used to evaluate goodness-of-fit.

We used the likelihood ratio test (Δχ^2^/Δdf) as a method of comparing model fit^53^. We also calculated changes in RMSEA (ΔRMSEA) relative to the parent model to compare models more easily^54^. A description and rationale for all model evaluation methods is available in Supplementary Material 4.1.

##### Tests of reliability and validity

Internal consistency and construct reliability were assessed using McDonald’s ω and 95% CI, both at a factor-level and a scale-level, for the model with the best fit. We used ω instead of Cronbach’s α (alpha) since the assumption of tau-equivalence was violated in our case^55^. Test–retest reliability was assessed using Spearman’s rho (ρ), intra-class correlation (ICC) and inspection of Bland–Altman plots. The employment of Spearman's rho was informed by the results of the Shapiro–Wilk test, which indicated a non-normal distribution of the total PPS scores (Shapiro–Wilk statistic = 0.967, p < 0.001). This finding was visually corroborated through an examination of the distribution, as evidenced by histograms and Q-Q plots (refer to Section A 4.3 in the Supplementary Material). Bland–Altman plots were used to visually assess the disagreement between the measurements on two different measurement days (Supplementary Material A4.2).

The construct validity of the PPS was evaluated by inspecting how total and subscale PPS scores correlated with a separate, single-item measure of overall preparedness [“*Please [drag the slider to] describe how prepared you felt overall for your psychedelic experience* (1 = not at all prepared; 5 = completely prepared)”]. Convergent and divergent evidence was evaluated by inspecting the correlations between PPS scores and validated measures that were expected to be positively correlated with psychedelic preparedness (e.g. EBI, MEQ), and measures that were expected to be negatively correlated with psychedelic preparedness (e.g. CEQ). Discriminant validity was evaluated by inspecting the correlations between PPS scores and validated measures that were expected to be unrelated to psychedelic preparedness (e.g. ECR-SAn, BFI-Ex). Finally, the criterion validity (i.e. predictive validity) was assessed by inspecting how total PPS scores correlated with total scores on the ‘preparation efficacy’ measure comprising 10 items, and by examining whether the total PPS scores correlated with changes in three separate outcomes (post-psychedelic growth (PPGI), centrality of event (COES) and perceived change in well-being as a consequence of the psychedelic experience (SWEMWBS)) using data from.

To further demonstrate the impact of psychedelic preparation level on outcomes, participants with PPS total scores below the median were categorised as 'Low Preparation' and those with PPS total scores above the median were categorised as 'High Preparation'. Two-sample t-tests were used to compare the means of each relevant outcome measure (EBI, MEQ, CEQ, PPGI, COES, SWEMWBS) between the two groups.

### Results

#### Demographic

The demographic characteristics of Sample A and B participants can be found in Supplementary Material A5.1

#### Exploration of factor structure

All 41 original preparedness items (excluding the 10 preparation efficacy items) from Sample A dataset (N = 518) were entered into a maximum likelihood based EFA. Sample A was found to be suitable for factor analysis, as its KMO measure of sampling adequacy was 0.913 and Bartlett’s test of sphericity was significant (χ^2^(820) = 11,257.648, p < 0.001)^56^. Visual inspection of the scree plot (Supplementary Material A6.1), parallel analysis and optimal coordinate estimates suggested a 4-factor solution, which was chosen for the subsequent extraction of factor loadings. Item loading patterns (Supplementary Material A6.2) revealed that 14 items had significant cross loadings (> 0.2), and 7 items had factor loadings less than 0.30, which were therefore removed and EFA repeated (Supplementary Material A6.3).

The remaining 20 items formed the final ‘psychedelic preparedness scale’ (Supplementary Material B), and all had satisfactory loading patterns, where the first factor explained 16.04% of the variance, the second factor 15.65%, the third factor 12.51%, and the fourth factor 12.33%, amounting to a total of 56.53% of variance explained in a 4-factorial solution. Based on the content of items that loaded on each factor, appropriate labels were assigned that each represented a sub-domain of psychedelic preparedness. Accordingly, the four factors were named ‘Knowledge-Expectations’ (K-E), ‘Intention-Preparation’ (I-P), ‘Psychophysical-Readiness’ (P-R), and ‘Support-Planning’ (S-P).

#### Test of dimensionality

**Figure 2:**
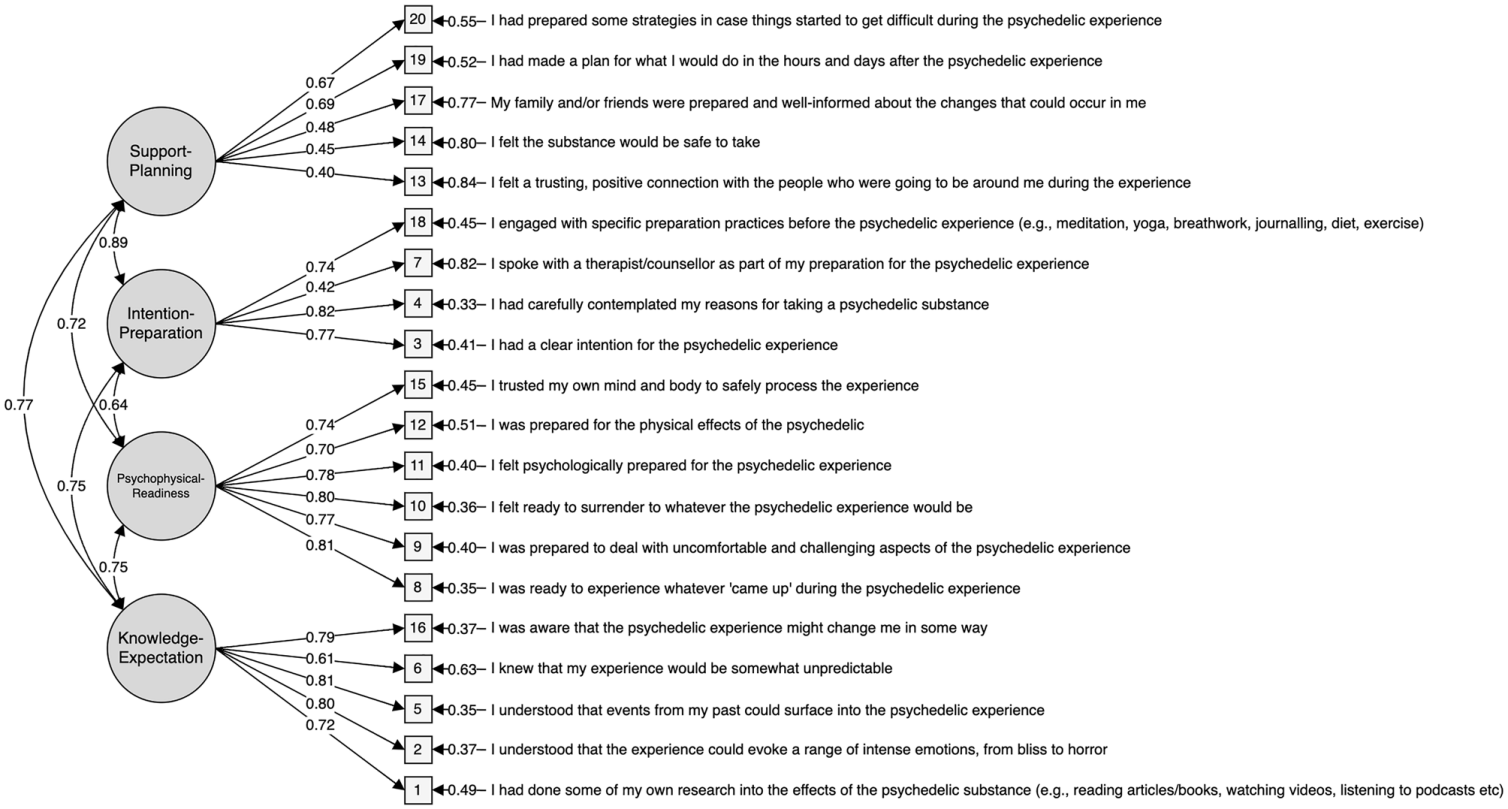
Structural representation of PPS Model 1. CFA Confirmatory factor analysis model with standardised loadings. Included in the model (N = 718) were 20 items of the PPS which fulfilled loading criteria during exploratory factor analyses in a different sample (N = 518). Circles represent latent variables, squares represent observed indicators, one-way arrows represent paths and two-way arrows represent covariances.

The 4-factor structure (Model 1) was then subjected to a subsequent confirmatory factor analysis (CFA) in the separate sample (Sample B) dataset (N = 718). Although the chi-square test was significant for Model 1 (χ^2^ (66, N = 718) = 611.973, p < 0.001))^51,52^ other fit indices suggested acceptable fit with CFI = 0.936, TFI = 0.925, RMSEA = 0.062 (CI 0.057–0.067) and SRMR = 0.054. Standardised factor loadings of the resulting model are shown in Fig. 2.

CFA was also conducted on three nested models which were compared to the hypothesised 4-factor model (Table 2). Models 2 and 3 showed acceptable fit. However, the hypothesised 4-factor model showed lowest RMSEA values and a lower likelihood ratio test (χ2/df) result relative to all other models, which was considered more favourable^53,54^ (Supplementary Material A4.1).

**Table 2:** Model fit indices for nested structural models of the PPS.

	Model	RMSEA (95% CI)	SRMR	CFI	TLI	Χ^2^ (df)	ΔΧ^2^Δdf^a^	ΔRMSEA^b^
1	**4-factor model**	**0.062 (0.057–0.067)**	**0.054**	**0.936**	**0.925**	**611.973 (164)**	–	
2	Hierarchical 4-factor model	0.064 (0.059–0.069)	0.055	0.929	0.919	658.682 (166)	+ 46.709/ + 2	+ 0.002
3	3-factor model	0.064 (0.058–0.069)	0.059	0.930	0.921	651.600 (167)	+ 39.627/ + 3	+ 0.002
4	2-factor model	0.082 (0.077–0.087)	0.061	0.883	0.869	982.354 (169)	+ 370.384/ + 5	+ 0.020

The model with the best fit is shown in bold. N = 718. RMSEA, root mean square error of approximation; CI, confidence interval; SRMR, standardised root mean squared residual; CFI, Comparative Fit Index; TFI, Tucker-Lewis Fit Index; χ^2^, Chi-square; df, degrees of freedom.

^a^Likelihood ratio test, shown as a change in χ^2^/df values relative to the parent model i.e. the hypothesised 4-factor model (top row).

^b^Change in RMSEA value relative to the parent model i.e. the hypothesised 4-factor model (top row).

#### Reliability

The 20 items from the PPS showed excellent total scale reliability (McDonald’s omega (ω) = 0.945, 95% CI  0.940–0.949 ), as did all sub-scales in the hypothesised 4-factor model: K-E (ω = 0.890, 95% CI   0.880–0.900 ), I-P (ω = 0.865, 95% CI   0.854–0.877), P-R (ω = 0.828, 95% CI   0.813–0.844), and S-P (ω = 0.799, 95% CI   0.782–0.817) (Supplementary Material A7.1).

**Figure 3:**
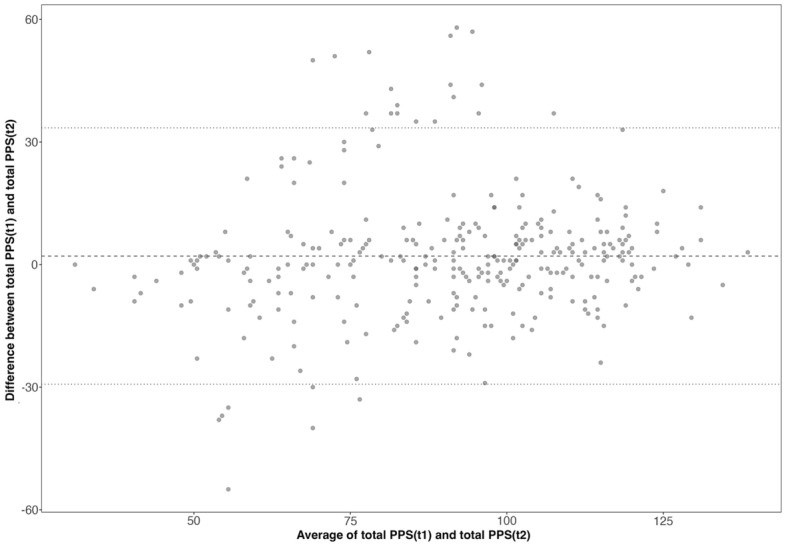
Test–retest reliability Bland–Altman plot. Intraindividual differences (n = 296) between mean PPS scores for test–retest, plotted against the average of the two scores. The central line represents the mean difference, and the top and bottom lines display the 95% confidence interval.

A sub-sample (N = 296) from sample A was used to estimate the test–retest reliability of the PPS. The Bland–Altman plot was used to visually inspect the test–retest scores. As displayed in Fig. 3, 25 (8.45%) of the 296 within-subject test–retest difference scores were outside of the 95% CI [− 29.28, 33.458]. There was a mean difference (t1-t2) on total PPS scores of 2.091 between test occasions (95% CI   0.261–3.922). The test and retest reliability metrics in these 296 participants suggested good reliability (ρ = 0.77, p < 0.001, ICC = 0.76). Test–retest reliabilities for the four separate factors were also indicative of good reliability: K-E (ρ = 0.77, p < 0.001, ICC = 0.79), I-P (ρ = 0.77, p < 0.001, ICC = 0.74), P-R (ρ = 0.77, p < 0.001, ICC = 0.62), and S-P (ρ = 0.77, p < 0.001, ICC = 0.70).

#### Validity

**Figure 4:**
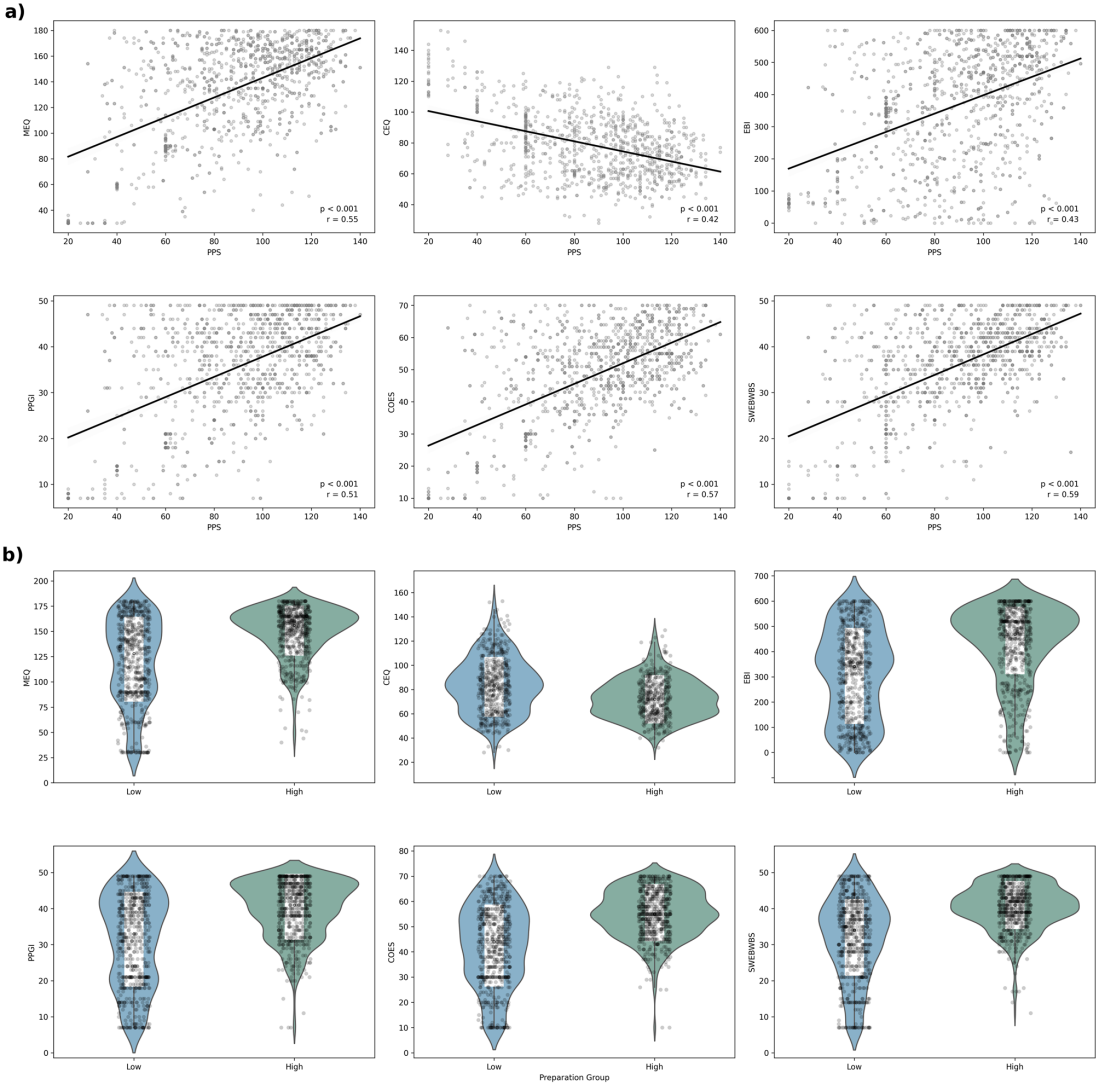
Retrospective PPS predicts quality of experience and therapeutic outcomes. Panel (**a**) shows scatter plots illustrating the correlation between total PPS scores and MEQ (r = 0.55, p < 0.001), CEQ (r = − 0.42, p < 0.001), EBI (r = 0.43, p < 0.001), PPGI (r = 0.51, p < 0.001), COES (r = 0.57, p < 0.001) and SWEBWBS (r = 0.59, p < 0.001). Panel (**b**) displays violin plots for group differences between high and low preparation groups for all six outcomes. The high preparation group scored significantly higher on the MEQ (t(1234) = 14.654, p ≤0.001, EBI (t(1234) = 11.945, p ≤ 0.001), PPGI (t(1234) = 14.437, p ≤ 0.001), COES (t(1234) = 17.235, p ≤ 0.001), and SWEBWBS (t(1234) = 16.663, p ≤ 0.001), and lower scores on the CEQ (t(972) = − 8.647, p ≤ 0.001) than the low preparation group.

In Sample A (N = 518), total PPS scores were moderately (positively) correlated with the single item rating of overall preparedness (Spearman’s rho = 0.55, p < 0.001), supporting construct validity of the multivariate PPS. In the pooled sample (N = 1236), correlation coefficients between total PPS, its subscales and other validated measures are displayed in Table 3, demonstrating convergent and discriminant evidence. Results revealed a strong positive correlation between the total PPS score and the overall preparation efficacy score (r = 0.81, p < 0.001). PPS totals were also moderately correlated with all three ‘criterion’ variables (PPGI: (r = 0.51, p < 0.001, COES: r = 0.57, p < 0.001, SWEMWBS: r = 0.59, p < 0.001), demonstrating criterion validity (see Fig. 4a for relevant scatterplots).

**Table 3:** PPS convergent, divergent and discriminant validity.

					Convergent		Divergent^b^	Discriminant^a^	
	PPS total	KE	IP	PR	MEQ	EBI	CEQ	ECR-SAn	BFI-Ex
PPS total	–				0.55*	0.43*	− 0.42*	− 0.05	0.01
KE	0.87*	–			0.55*	0.48*	− 0.41*	− 0.03	0.16
IP	0.88*	0.73*	–		0.57*	0.42*	− 0.49*	− 0.01	0.24
PR	0.82*	0.69*	0.81*	–	0.55*	0.43*	− 0.41*	− 0.06	0.01
SP	0.82*	0.82*	0.61*	0.71*	0.53*	0.45*	− 0.41*	− 0.02	0.05

Correlation coefficients between the PPS, total and PPS subscales scores and validated secondary measures. Coefficients are Pearson’s r.

*Sig at p < 0.001. *KE*   knowledge-expectations, *IP*  intention-preparation, *PR*   psychophysical-readiness, *SP* =  support-planning.

^a^Data for these surveys was only collected in Sample B (N = 718).

^b^Data for this measure was collected from all 518 participants in Survey A and 457 participants in Survey B, making a total sample of N = 975.

Descriptive statistics for high and low preparation groups on the MEQ, CEQ, EBI, PPGI, COES, and SWEBWBS outcomes can be found in Supplementary Material A8.1. Independent t-tests were conducted to compare the mean scores of the high (upper 50th %ile) and low (lower 50th %ile) preparation groups on each outcome. Results showed significant differences between the high and low preparation groups on all six criterion variables (Fig. 4b). Specifically, the high preparation group had significantly higher mean scores on the MEQ (t(1234) = 14.654, p ≤ 0.001, EBI (t(1234) = 11.945, p ≤ 0.001), PPGI (t(1234) = 14.437, p ≤ 0.001), COES (t(1234) = 17.235, p ≤ 0.001), and SWEBWBS (t(1234) = 16.663, p ≤ 0.001), and lower scores on the CEQ (t(972) = − 8.647, p ≤ 0.001) than the low preparation group.

## Study 3: preliminary scale implementation

### Methods

#### Procedures

To assess the PPS as a prospective measure of psychedelic preparedness (i.e. before a planned psychedelic experience), it was administered to a sample [N = 46] of participants before (− 1 day) they participated in a psilocybin ceremony (see Supplementary Material B1.1 for prospective PPS). It was repeated after (+ 2 weeks) the ceremony to replicate the reliability findings reported in Study 2. In addition to completing the PPS, participants also completed the 11D-ASC and DASS-21 to further examine criterion validity.

#### Recruitment and data collection

Participants (N = 46) were recruited through collaborating retreat centres who invited participants enrolled on 5–7-day residential psilocybin retreats to join the study prior to their arrival. To be eligible, participants needed to be willing to complete the surveys before and after the retreat. Participants were included in the data analysis if they fully completed all relevant questionnaires at both timepoints.

#### Data analysis

##### Reliability and validity

Internal consistency and construct reliability were assessed as above (Study 2).

Correlations were used to determine whether the prospective total PPS scores were associated with the global-ASC scores (average score over all dimensions), and changes in the three subscale scores of the DASS scores (criterion validity).

To further demonstrate the impact of psychedelic preparation level on outcomes, the data was split into two groups of high and low preparation (using the same methods as Study 2) and two-sample t-tests were used to compare the global-ASC scores, means of each 11D-ASC subscale, and change in DASS subscale scores between the two groups.

### Results

#### Demographic

The demographic characteristics of participants in the Retreat Survey can be found in Supplementary Material A9.1.

#### Reliability

The retrospective PPS scores were used to estimate the test–retest reliability of the prospective PPS scores. The Bland–Altman plot method was used to visually inspect the test–retest reliability after 2 weeks (Supplementary Material A10.1). Similar to the results obtained in Study 2, 6.52% (3/46 participants) of the within-subject test–retest difference scores were outside of the 95% CI [− 22.62, 28.32]. There was a mean difference (t1–t2) of 2.848 between the days (95% CI   − 1.01, 6.71). Spearman’s rho (ρ) and intraclass correlation coefficients (ICC2,1) obtained in this smaller ecological sample were also similar to those reported for Study 2 for the total PPS (ρ = 0.71, p < 0.001, ICC = 0.71) and the sub-domains of preparation: : K-E (ρ = 0.71, p < 0.001, ICC = 0.81), I-P (ρ = 0.71, p < 0.001, ICC = 0.62) P-R (ρ = 0.71, p < 0.001, ICC = 0.58), and S-P(ρ = 0.71, p < 0.001, ICC = 0.54).

#### Validity

**Figure 5:**
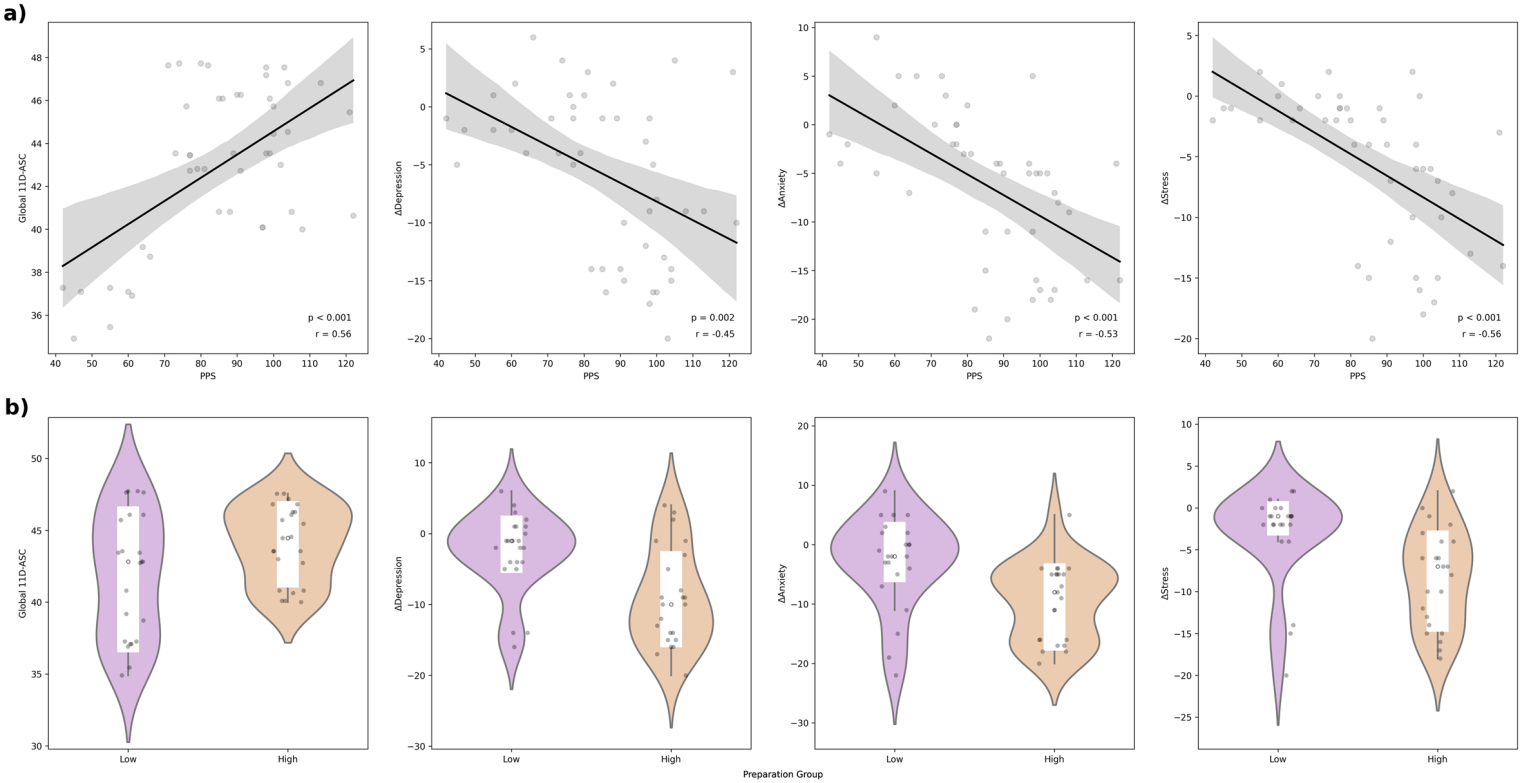
Prospective PPS predicts quality of experience and therapeutic outcomes. Panel (**a**) displays scatter plots illustrating the correlations between total PPS scores and global-ASC (r = 0.56, p < 0.001), and changes in the DASS subscales (depression: r =  − 0.45, p = 0.002; anxiety: r =  − 0.53, p < 0.001; stress: r =  − 0.56, p < 0.001). Panel (**b**) shows violin displays violin plots for group differences between high and low preparation groups for all four outcomes. The high preparation group scored significantly higher on the global-ASC (t(44) = 2.106, p = 0.041), and had significantly greater reductions in Depression (t(44) =  − 3.526, p =  < 0.001, Anxiety (t(44) =  − 3.202, p = 0.003), and Stress (t(44) =  − 3.162, p = 0.003) scores, than the low preparation group.

Total PPS score was significantly correlated with global-ASC score (r = 0.56, p < 0.001), and changes in total DASS scores (depression: r = − 0.0.45, p = 0.002; anxiety: r = − 0.53, p < 0.001; stress: r = − 0.56, p < 0.001) (Fig. 5a).

Results showed significant differences between the high (upper 50th %ile) and low (lower 50th %ile) preparation groups on global-ASC score (t(44) = 2.106, p = 0.041) (Fig. 5b), and eight of the eleven 11D-ASC outcomes (Supplementary Material A11.1). Specifically, the high preparation group had significantly higher mean scores on the experience of unity (t(44) = 4.719, p ≤ 0.001, spiritual experience (t(44) = 3.545, p ≤ 0.001), blissful state (t(44) = 3.117, p = 0.003), insightfulness (t(44) = 3.358, p = 0.002), disembodiment (t(44) = 4.375, p ≤ 0.001), and lower mean scores on the anxiety (t(44) = − 2.348, p = 0.023) and elemental imagery (t(44) =  − 3.432, p = 0.001) than the low preparation group (see Supplementary Material A 11.2 for radar plot).

Results showed significant differences between the high and low preparation groups on change scores of all three measures of psychological distress from the DASS-21 (Fig. 5b). Specifically, the high preparation group had significantly greater reductions in Depression (t(44) = -3.526, p =  < 0.001, Anxiety (t(44) = -3.202, p = 0.003), and Stress (t(44) = -3.162, p = 0.003) scores, than the low preparation group (Supplementary Material A11.2).

## Discussion

This article introduces and validates the Psychedelic Preparedness Scale (PPS), a new self-report measure. In Study 1, our novel 'DelFo' method was used to develop the questionnaire items, and subsequent QPIs were employed to reach consensus on structure and content. In Study 2, the 20-item PPS demonstrated good reliability and validity and was able to predict both acute and post-acute psychological effects induced by psychedelics. Study 3 further confirmed these findings, revealing the PPS to be an effective prospective as well as retrospective tool for measuring preparedness and estimating responses to psychedelics (see Supplementary Material B for prospective and retrospective PPS).

The results of our studies provide compelling evidence for the existence of a measurable psychological state of psychedelic preparedness, which is predictive of positive acute and long-term outcomes associated with psychedelic use. Our study utilised robust scale development and psychometric validation methodology, revealing four distinct underlying factors of psychedelic preparedness: Knowledge-Expectations, Intention-Preparation, Psychophysical-Readiness, and Support-Planning. The 4-factor model of the Psychedelic Preparedness Scale (PPS) exhibited superior goodness of fit in comparison to the second-order model; nonetheless, the hierarchical model still demonstrated adequate fit and may offer enhanced clinical convenience for implementation in research and clinical settings. Notably, the hierarchical model provides simplified scoring, interpretation, and representation of the overarching constructs, along with flexibility in its application. For instance, the PPS could serve as a valuable screening tool to identify individuals who may require additional preparation or support prior to undergoing psychedelic interventions, thereby functioning as a pre-intervention assessment to pinpoint areas of preparedness that necessitate attention. Furthermore, the PPS could serve as an outcome measure to evaluate the effectiveness of psychedelic preparedness interventions or to compare the efficacy of different preparation protocols. As such, future research endeavours should continue to explore the potential applications of the PPS and its role in the development of individualised treatment plans in clinical settings, as well as investigate the relative influence or interaction of each factor in predicting acute and long-term effects of psychedelics.

The present study makes a unique and comprehensive contribution to the field in part by employing a methodology that emphasises Patient and Public Involvement (PPI). PPI is a critical but often overlooked aspect of measure development processes^57–59^. However, despite challenges posed by paternalistic beliefs that may hinder collaboration with individuals who have lived experience^18^, involving end-users in research activities can empower these individuals, foster trust in the research, and optimise the research process^60^. When done systematically and thoughtfully, PPI can significantly enhance the credibility and impact of research, promoting accountability, transparency, and relevance^61^. Despite the establishment of recent guidelines for PPI strategy in psychedelic science^62^, the lack of patient/public collaborators acknowledged in this field is still evident. In this paper, we aimed to address this issue by incorporating PPI into our scale development process, allowing us to test clinical and theoretical tenets of psychedelic preparedness^9,16^, while also identifying gaps in our understanding of the acceptability and feasibility of the scale, among end-users who would actually be using it^60,63^. This approach aligns with the principles of Community-Based Participatory Research (CBPR), which promotes equitable partnerships between researchers and communities, emphasising the value of local knowledge and expertise^64^. Accordingly, the consensus-focused ambition of this paper, which incorporated lived-experience perspectives in a co-production fashion, represents a significant contribution to the field of psychedelic research.

There are several limitations to this study. Firstly, the validation of the PPS is limited by a self-selection bias for all three studies. Study participants were predominantly WEIRD (white, educated, industrialised, rich, democratic)^65^ reflecting a pervasive problem in psychedelic research^66^. We encourage future studies to conduct a validation and analysis of PPS scores in ethnically, geographically, and culturally diverse settings. Secondly, the high attrition rate in the retreat study meant only 46 participants completed the before and after surveys, which could have created attrition bias effects. Participants who dropped out of the study may have had different experiences and outcomes compared to those who completed the study, potentially affecting the results. It is important for future research to address potential attrition bias by carefully monitoring and accounting for participant dropout rates. Furthermore, it should be noted that while participants in the survey studies were permitted to have used a variety of psychedelics, the retreat studies exclusively focused on psilocybin. This limitation may restrict the generalizability of the findings to other types of psychedelics, as there could be potential variations in the impact of preparation on acute and long-term effects depending on the specific substance used. Lastly, the present article provides no specific examination of the validity and clinical utility of the PPS in and between different clinical populations. To address these issues, future research should systematically investigate the role of preparedness for different types of psychedelics in different neuropsychiatric conditions, allowing for a more comprehensive understanding of potential between-psychedelic effects and their implications.

In sum, despite the current lack of standardisation in psychedelic preparatory sessions^67^ and limited research on measuring or enhancing this critical pre-treatment state^9^, our findings support the notion that optimising this multi-factorial state should be a crucial consideration in the design of any scenario involving the ingestion of psychedelic drugs. However, we emphasise that the PPS should not be utilised as a standalone diagnostic tool for making clinical decisions, but rather integrated with other comprehensive clinical assessments as part of a comprehensive treatment plan.

## Conclusion

The results of this study provide preliminary evidence that positive preparatory context is associated with greater therapeutic change. The development of the PPS represents an important step in defining and elucidating the consequences of pre-treatment factors. By providing a reliable and valid measure of preparedness, the PPS may facilitate the development of evidence-based preparatory interventions that enhance the safety and therapeutic potential of psychedelics, while contributing to a deeper understanding of the mechanisms by which psychedelics acutely and longitudinally alter human experience.

### Supplementary Information

Supplementary Information.

## Data Availability

The data generated and/or analysed during this research will be made available upon request by contacting the corresponding author.
