# Supplementary material for: Development and psychometric validation of a novel scale for measuring ‘psychedelic preparedness’
Source: Sci Rep. 2024 Feb 8;14:3280. doi: 10.1038/s41598-024-53829-z (PMC10853197; doi:10.1038/s41598-024-53829-z)
Supplement: Supplementary file 1 — Supplementary Information. [file 41598_2024_53829_MOESM1_ESM.pdf]

Supplementary Material A
Title: Development and psychometric validation of a novel scale for measuring ‘psychedelic preparedness’.
Authors: Rosalind G. McAlpine[1*], George Blackburne[1,2], and Sunjeev K. Kamboj[1]

1.1 Adapted PGTI-SF

The next set of questions ask about the impact of your most significant psychedelic experience and the extent to which you believe things have changed in your life because of that experience. Please indicate how much you agree or disagree with the following statements (0: Strongly disagree; 7: Strongly agree)

1. The experience has changed who i am
2. The experience has become a reference point for the way I understand myself and the world.
3. The experience has become a central part of my life story.
4. The experience has coloured the way I think and feel about other experiences.
5. The experience permanently changed my life.
6. I often think about the effects the experience will have on my future.
7. The experience was a turning point in my life.

1.2 Adapted COES

The next set of questions ask about the impact of your most significant psychedelic experience and the extent to which you believe things have changed in your life because of that experience (0: Strongly disagree; 7: Strongly agree).

Since my most significant psychedelic drug experience…

1. I have changed my priorities about what is important in life.
2. I have a greater appreciation for the value of my own life.
3. I have a better understanding of spiritual matters.
4. I have established a new path for my life.
5. I have a greater sense of closeness with others.
6. I am better able to handle difficulties.
7. I am able to do better things with my life.
8. I have a stronger religious faith.
9. I discovered that I'm stronger than I thought I was.
10. I learned a great deal about how wonderful people are.

2.1 Demographic characteristics of Delphi participants ('expert judges')

		Round 1 (n = 12)	Round 2 (n = 10)	Round 3 and 4 (n = 7)
**Gender**	Male	7 (58.3%)	6 (60%)	4 (57.1%)
	Female	5 (44.7%)	4 (40%)	3 (42.9%)
**Mean age in years (SD)**		42.08 (7.34)	42.30 (7.97)	43.71 (7.89)
**Country of residence**	UK	4 (33.3%)	2 (20%)	2 (28.6%)
	USA	4 (33.3%)	4 (40%)	3 (42.9%)
	Netherlands	2 (16.7%)	2 (20%)	1 (14.3%)
	Australia	1 (8.3%)	1 (10%)	0 (0%)
	Germany	1 (8.3%)	1 (10%)	1 (14.3%)
**Current role**	Professor	5 (44.7%)	3 (30%)	2 (28.6%)
	Associate Professor	1 (8.3%)	1 (10%)	0 (0%)
	Lecturer	1 (8.3%)	1 (10%)	1 (14.3%)
	Psychiatrist [a]	3 (25%)	3 (30%)	2 (28.6%)
	Clinical Psychologist [a]	2 (16.7%)	2 (20%)	2 (28.6%)
**Years working in the field**	10+ years	7 (58.3%)	6 (60%)	4 (57.1%)
	6-9 years	3 (25%)	2 (20%)	1 (14.3%)
	4-5 years	1 (8.3%)	1 (10%)	1 (14.3%)
	1-3 years	1 (8.3%)	1 (10%)	1 (14.3%)

[a] *Non-clinical professionals*

2.2 Demographic characteristics of focus group participants (‘participant judges’)

**Gender (n = 6)**	Male	3 (50%)
	Female	3 (50%)
**Mean age in years (SD)**		44 (7.21)
**Ethnicity**	White	5 (83.3%
	Latin American	1 (16.7%)
**Country of residence**	UK	2 (33.3%)
	USA	2 (33.3%)
	Portugal	1 (16.7%)
	Hawaii	1 (16.7%)
**Education Level**	Undergraduate Degree	1 (16.7%)
	Master's Degree	2 (33.3%)
	Doctorate Degree	3 (50%)
**Experience with PAP**	Psilocybin for depression trial participant	3 (50%)
	Psilocybin for alcohol use disorder trial participant	1 (16.7%)
	Psilocybin retreat participant	1 (16.7%)
	Underground PAP client	1 (16.7%)

2.3 Demographic characteristics of qualitative pretest interview participants

**Gender (n = 6)**	Male	4 (66.7%)
	Female	2 (33.3%)
**Mean age in years (SD)**		36.5 (9.69)
**Ethnicity**	White	5 (83.3%
	Mixed	1 (16.7%)
**Country of residence**	UK	2 (33.3%)
	USA	2 (33.3%)
	Netherlands	1 (16.7%)
	Germany	1 (16.7%)
**Education Level**	Undergraduate Degree	3 (50%)
	Master's Degree	2 (33.3%)
	Doctorate Degree	1 (16.7%)
**Experience with PAP**	Attended a 5-day psilocybin retreat in Netherlands with 2 dosing sessions	3 (50%)
	Attended a 5-day psilocybin retreat in Mexico with 2 dosing sessions	1 (16.7%)
	Attended a 7-day psilocybin retreat in Mexico with 2 dosing sessions	2 (33.3%)

3.1 Examples of QPI informed item modifications. Prospective adaptations are presented in brackets.

Domain	Original Item	QPI Response	Updated Item
**Expectation-management**	I had (have) realistic expectations for the psychedelic experience	*P2: "I'm thinking back to what I expected of the experience, mainly in the weeks leading up to it, and I don't know if [I] really understand what a realistic expectation is. Does this mean my expectations became in line with my actual reality or does it mean they were suitable and not too far-fetched, and they possibly could have become my reality... I also feel like it slightly trips me up because I really have to think back to the, almost, accuracy or good-ness of those expectations and then reality check them. Basically, er, I think this is a bit confusing."*	My expectations for the experience were (are) accurate
		P3: "As I'm reading this out loud, the word realistic… it seems sort of jarring. I don't really know what it would mean to be realistic, and I don't feel immediately as if I'm in the position to assess the realistic-ness of these expectations or the experience. It just, well, I feel like something simpler about expectations matching experience would be better. If that's what you're asking."	
**Psychological mindedness**	I went into (am going into) the psychedelic experience with a willingness to try and understand myself more	*P2: "I'm not really sure if the word try works here. It makes me wonder how hard I tried to do this back then, and I get a bit caught up in this. So, [it] becomes kinda meaningless for me. I can't really tell if I was trying, or if I was trying hard enough. I do think I tried this, but if you asked me something, like, if I was willing and open, I'd be able to answer more easily."*	I went into (am going into) the experience willing to learn more about the meaning behind my thoughts
		P4: "I get what this is saying but the phrase willingness to try and understand seems a bit odd. Does this mean I was willing to do something, or a willingness to try and understand, or that I really was able to understand. Just because I'm willing to try and understand something, does this then impact whether or not I then understood it. I feel like I get a bit caught up in working out what this means."	

**Willingness to surrender**	I was (am) willing to accept whatever 'came up' ('comes up') during the psychedelic experience	*P1: "It's hard for me to remember whether or not I was capable [of] saying before the experience that I could accept whatever came up during it, but I do get what this question is trying to ask. I just don't know if before the experience I could really make claims like this, because the accepting bit is sometimes pretty hard. I don't think I would want to accept everything during the experience. That kind of takes all of your autonomy away from [it]. I don't think it's just about accepting."*	I was (am) ready to experience whatever 'came up' ('comes up') during the psychedelic experience
		P5: "Accept is pretty loaded in this statement. It makes me feel slightly as though I've given up on something - in a weird way. You could try shifting the word 'experience', so 'I was willing to experience' and then whatever came up for me."	
		P5: "Er, well, I feel like if you've decided and consented to taking the drug, you are kind of agreeing and expecting things to come up associated with that drug. You've chosen to take this drug so this kind of implies you're willing to allow the drug to take its effects. Maybe for some people they took the drug and then didn't want anything to come up, like, maybe they wanted a funny nice time with friends. But I just think it's a bit confusing because… surely, you're going to take the drug knowing that these things will come up. It's also really ambiguous like I'm not really sure what 'come up' even means, differentiated from allowing the drug to just do its thing."	
**Prepared for change**	I feel as though the psychedelic experience has permanently changed me	*P3: "This one is tricky because I can't really tell if I've been permanently changed. I feel different and I can track certain changes. But erm, I feel this is too broad. Especially if we are trying to measure this within one single item."*	I correctly anticipated the kinds of changes that occurred

		P6: "The first thing that comes to mind is the question of what is 'me', like, can I really describe 'me' in a single way. I'm thinking about certain qualities I possess and I feel as though I am made up of various parts. I do feel as though my psychedelic experience changed parts of me, but I still feel like, at the core, I am me."	

3.2 51-items (11 subdomains) selected at the end of the DelFo rounds and QPIs

	Retrospective	Prospective
**Psychoeducation**	I had done some of my own research into the effects of the substance (e.g., reading articles/books, watching videos, listening to podcasts etc)	I have done some of my own research into the effects of the substance (e.g., reading articles/books, watching videos, listening to podcasts etc)
	I had learned about the effects of the substance through conversations with other people	I have learned about the effects of the substance through conversations with other people
	I understood that the experience could evoke a range of intense emotions, from bliss to horror	I understand that the experience might evoke a range of intense emotions, from bliss to horror
	I was completely unaware of what to expect from the experience	I am completely unaware of what to expect from the experience
	I should have known more about the substance I was going to take	I should know more about the substance I am going to take
	My prior research and knowledge about the psychedelic were crucial to what I experienced (PE)	-
**Intention Setting**	I had a clear intention for the psychedelic experience	I have a clear intention for the psychedelic experience
	I was sure that keeping my intention in mind would help guide the psychedelic experience	I am sure that keeping my intention in mind will help guide the psychedelic experience
	I had carefully contemplated my reasons for undergoing the psychedelic experience	I have carefully contemplated my reasons for undergoing the psychedelic experience
	My intentions influenced my experience (PE)	-
**Expectation Management**	I expected the psychedelic experience to completely change my life	I expect the psychedelic experience to completely change my life
	I was expecting the psychedelic experience to be easy and fun	I expect the psychedelic experience to be easy and fun
	I understood that events from my past could surface into the psychedelic experience	I understand that events from my past might surface into the psychedelic experience
	I knew that my experience would be somewhat unpredictable	I know that my experience will be somewhat unpredictable
	My expectations for the experience were accurate (PE)	-
**Psychological Mindedness**	I spoke with a therapist/counsellor as part of my preparation for the psychedelic experience	I have spoken with a therapist/counsellor as part of my preparation for the psychedelic experience
	I went into the experience willing to learn more about the meaning behind my thoughts	I am going into the experience willing to learn more about the meaning behind my thoughts
	I went into the experience with a curiosity about my own mind	I am going into the experience with a curiosity about my own mind

	Engaging with my thoughts/feelings in the lead up to my experience impacted the experience itself (PE)	-
**Emotional Readiness**	I felt ready for the psychedelic experience	I feel ready for the psychedelic experience
	I had serious doubts before going into the psychedelic experience	I have serious doubts about going into the psychedelic experience
	I felt afraid going into the psychedelic experience	I feel afraid about going into the psychedelic experience
	I felt comfortable going into the psychedelic experience	I feel comfortable about going into the psychedelic experience
	I felt emotionally ready for what I experienced (PE)	-
**Willingness to Surrender**	I was ready to experience whatever 'came up' during the psychedelic experience	I am ready to experience whatever 'comes up' during the psychedelic experience
	I was prepared to deal with uncomfortable and challenging aspects of the psychedelic experience	I am prepared to deal with uncomfortable and challenging aspects of the psychedelic experience
	I felt ready to surrender to whatever the psychedelic experience would be	I feel ready to surrender to whatever the psychedelic experience will be
	I was able to surrender during the experience (PE)	-
**Psychophysical Robustness**	I felt psychologically prepared for the psychedelic experience	I feel psychologically prepared for the psychedelic experience
	I was prepared for the physical effects of the psychedelic	I am prepared for the physical effects of the psychedelic
	I felt as though my mind and body would be 'strong enough' for the upcoming experience	I feel as though my mind and body will be 'strong enough' for the upcoming experience
	I was mentally and physically prepared for what I experienced while on the psychedelic substance (PE)	-
**Safety/Security**	In the lead up to the actual psychedelic experience, I felt a trusting, positive connection with the people around me	I feel a trusting, positive connection with the people who will be around me during the psychedelic experience
	I felt the substance would be safe to take	I feel the substance will be safe to take
	I trusted the quality and purity of the of the substance	I trust the quality and purity of the of the substance
	I trusted my own mind and body to safely process the experience	I trust my own mind and body to safely process the experience
	I felt safe and supported throughout my	-

	psychedelic experience (PE)	
**Prepared for Change**	I was aware that the psychedelic experience might change me in some way	I am aware that the psychedelic experience might change me in some way
	I felt ready to accept some big changes that might occur in myself as a result of the psychedelic experience	I feel ready to accept some big changes that might occur in myself as a result of the psychedelic experience
	My family and/or friends were prepared and well-informed about the changes that could occur in me	My family and/or friends are prepared and well-informed about the changes that might occur in me
	My friends and/or family were ready to support me through any changes I experienced	My friends and/or family are ready to support me through any changes I experience
	I was worried about becoming too different after the psychedelic experience	I am worried about becoming too different after the psychedelic experience
	I was afraid that I might change in a negative way after the psychedelic experience	I am afraid that I might change in a negative way after the psychedelic experience
	I correctly anticipated the kinds of changes that occurred (PE)	-
**Preparatory Practices**	I dedicated time to preparing for the psychedelic experience	I have dedicated time to preparing for the psychedelic experience
	I engaged with specific preparation practices before the psychedelic experience (e.g., meditation, yoga, breathwork, journaling, diet, exercise)	I have engaged with specific preparation practices in the lead up to the psychedelic experience (e.g., meditation, yoga, breathwork, journaling, diet, exercise)
	I planned out what I would do in the hours and days after the psychedelic experience	I have planned out what I will do in the hours and days after the psychedelic experience
	I told close/trusted friends and/or family that I was going to have the psychedelic experience	I have told close/trusted friends and/or family that I am going to have the psychedelic experience
	I didn't do anything to prepare for the psychedelic experience	I haven’t done anything to prepare for the psychedelic experience
	I had prepared some strategies in case things started to get difficult during the psychedelic experience	I have prepared some strategies in case things start to get difficult during the psychedelic experience
	My preparation impacted my experience (PE)	-

Note: Psychedelic Efficacy items (PE) were not included in the PPS.

4.1 Model evaluation methods

We used the likelihood ratio test ($\Delta\chi^2/\Delta df$, also called the χ^2 difference test) as a method of comparing between nested models, meaning those that have the same amount of items and differ in the number of factors i.e. latent variables (Brown, 2015). The hypothesized 4-factor model was the parent model to which all the other nested models were compared. At df=1, the critical value of χ^2 is 3.84 (α=.05), so a χ^2 difference of >3.84 for a change in 1df from parent to the (compared) nested model indicates that the parent model provides a significantly better fit (Brown, 2015). We also calculated changes in RMSEA (ΔRMSEA) relative to the parent model to compare models more easily.

4.2 Tests of reliability and validity

The ICC makes it possible to assess whether and to what extent systematic and random errors affect measurement repeatability; its possible values are in the range of 0.00–1.00 [43]. Reliability is thought to be poor if ICC values are lower than 0.40, fair if the values range from 0.40 to 0.59, good if the values are between 0.60 and 0.74, and excellent if the values are between 0.75 and 1.00 [44].

A plot of each participant's mean PPS score plotted against the patient score difference (PPS score in Survey A minus PPS score on Survey B) was constructed to check for possible systematic bias. The Bland–Altman plots displayed the 95% limits of agreement (95% LOA) which give a range within which it is expected the 95% of future differences in measurements between measurement days to lie. The 95% LOA was calculated as the difference in the mean scores of the test ± the score difference SD × 1.96.

4.3 Histograms and Q-Q plots illustrating the distribution of the total PPS scores

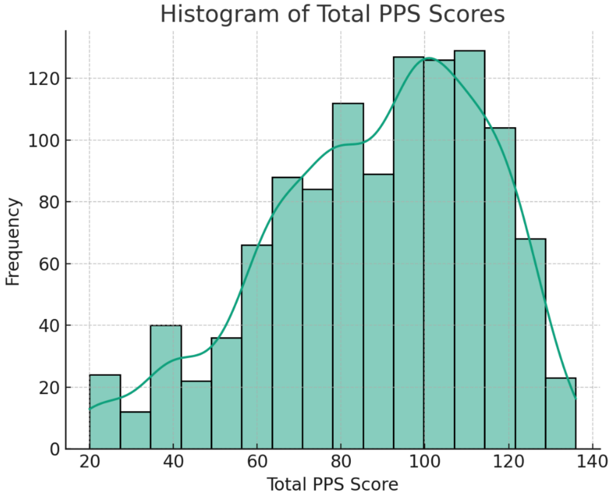

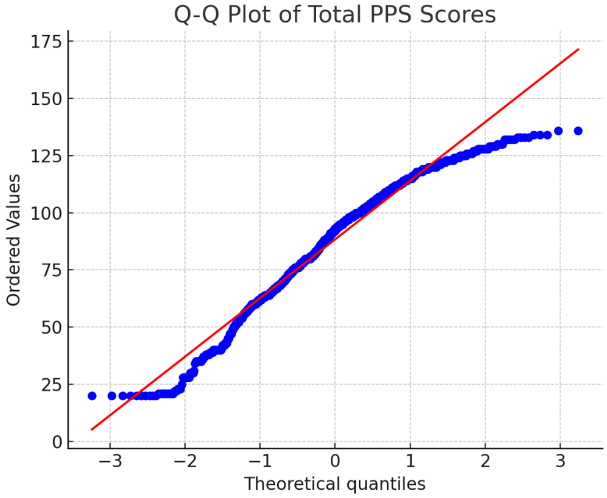

5.1 Demographic characteristics of Sample A and B

		Survey A<br>(N = 518)	Survey B<br>(N = 718)
**Gender**	Male	288 (55.6%)	314 (43.7%)
	Female	202 (39.0%)	372 (51.8%)
	Other	28 (5.4%)	32 (4.5%)
**Mean age in years (SD)**		33.72 (12.81)	37.24 (12.64)
**Lifetime psychedelic use**	On 1 occasion	33 (6.4%)	35 (4.9%)
	On 2-5 occasions	130 (25.1%)	139 (19.4%)
	On 6-10 occasions	103 (19.9%)	114 (15.9%)
	On 11-20 occasions	69 (13.3%)	127 (17.7%)
	On more than 20 occasions	183 (35.3%)	303 (42.2%)
**Religion**	Not religious	285 (55.1%)	293 (40.8%)
	Christian	45 (8.7%)	60 (8.4%)
	Islam	10 (1.9%)	9 (1.3%)
	Sikhism	2 (0.4%)	0 (0.0%)
	Secular	2 (0.4%)	n/a
	Judaism	9 (1.7%)	11 (1.5%)
	Hinduism	7 (1.4%)	9 (1.3%)
	Buddhism	21 (4.1%)	26 (3.6%)
	Spiritual	104 (6.2%)	379 (52.8%)
	Other	33 (0.1%)	31 (4.3%)
**Education**	Secondary/High School	136 (26.3%)	179 (24.9%)
	Undergraduate	169 (32.7%)	259 (36.1%)
	Postgraduate	212 (41.0%)	280 (39.0%)
**Ethnicity**	White	427 (82.8%)	578 (80.5%)
	Black	8 (5.4%)	14 (2.0%)
	Asian	28 (5.4%)	32 (4.4%)
	Other	55 (10.6%)	94 (13.0%)

6.1 Scree plot showing eigenvalues and results of non-graphical tests to determine optimal number of factors to extract from the PPA.

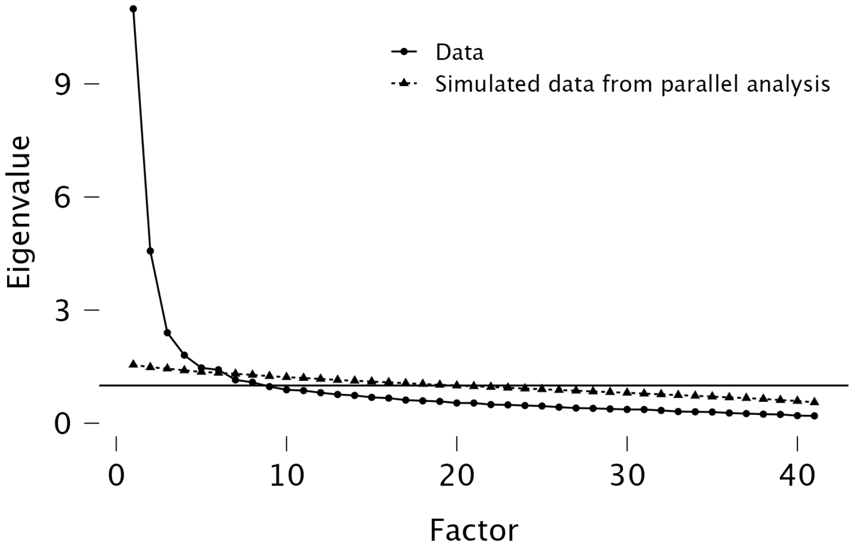

6.2 Item loading patterns (EFA)

**Factor Matrix**[a]				
	K-E	P-R	I-P	S-P
I had done some of my own research into the effects of the psychedelic substance (e.g., reading articles/books, watching videos, listening to podcasts etc)	0.72	0.02	0.18	-0.11
I had learned about the effects of the substance through conversations with other people	0.24	0.22	-0.14	-0.11
I understood that the experience could evoke a range of intense emotions, from bliss to horror	0.71	-0.09	0.02	-0.08
I was unaware of what to expect from the experience	0.60	0.22	0.10	-0.08
I should have known more about the substance I was going to take	0.52	0.29	0.17	-0.03
I had a clear intention for the psychedelic experience	0.04	0.15	0.64	-0.05
I was sure that keeping my intention in mind would help guide the psychedelic experience in a helpful direction	0.49	0.3	0.02	-0.11
I had carefully contemplated my reasons for taking a psychedelic substance	0.29	0.17	0.72	-0.23
I expected the psychedelic experience to change my life	0.07	0.21	0.22	0.02

I was expecting the psychedelic experience to be easy and fun	0.14	0.03	-0.07	0.29
I understood that events from my past could surface into the psychedelic experience	0.68	0.06	0.16	-0.05
I knew that my experience would be somewhat unpredictable	0.63	0.10	-0.09	-0.13
I spoke with a therapist/counsellor as part of my preparation for the psychedelic experience	0.18	-0.17	0.74	0.11
I went into the experience willing to learn more about the meaning behind my thoughts	0.11	0.27	0.51	0.01
I went into the experience with a curiosity about my own mind	0.18	0.27	0.51	0.2
I felt ready for the psychedelic experience	0.16	0.15	0.29	0.25
I had significant doubts before going into the psychedelic experience	0.23	0.06	0.43	0.17
I felt afraid going into the psychedelic experience	0.23	0.12	0.34	0.16
I felt comfortable going into the psychedelic experience	0.11	0.06	0.32	0.31
I was ready to experience whatever 'came up' during the psychedelic experience	0.03	0.66	0.12	0.14
I was prepared to deal with uncomfortable and challenging aspects of the psychedelic experience	0.12	0.65	0.14	0.01
I felt ready to surrender to whatever the psychedelic experience would be	0.18	0.65	-0.01	0.04
I felt psychologically prepared for the psychedelic experience	0.08	0.63	0.05	0.01
I was prepared for the physical effects of the psychedelic	0.11	0.62	0.15	0.08
I felt as though my mind and body would be 'strong enough' for the upcoming experience	0.10	0.68	0.32	0.02
I felt a trusting, positive connection with the people who were going to be around me during the experience	0.18	-0.17	0.13	0.74
I felt the substance would be safe to take	0.16	-0.13	0.14	0.68
I trusted the quality and purity of the of the substance	0.27	-0.2	0.21	0.67
I trusted my own mind and body to safely process the experience	0.04	0.58	0.04	-0.09
I was aware that the psychedelic experience might change me in some way	0.47	0.16	0.16	-0.01
I felt ready to accept some big changes that might occur in myself as a result of the psychedelic experience	0.25	-0.06	0.2	0.67
My family and/or friends were prepared and well-informed about the changes that could occur in me	-0.1	0.17	0.08	0.68
My friends and/or family were ready to support me through any changes I experienced	-0.26	0.21	0.10	0.25
I was worried about becoming too different as a person after the psychedelic experience	-0.09	-0.15	-0.25	0.33

I was afraid that I might change in a negative way after the psychedelic experience	0.26	0.19	0.25	0.33
I dedicated time to preparing for the psychedelic experience	0.10	0.11	0.25	0.29
I engaged with specific preparation practices before the psychedelic experience (e.g., meditation, yoga, breathwork, journalling, diet, exercise)	-0.12	0.01	0.73	0.20
I had made a plan for what I would do in the hours and days after the psychedelic experience	0.16	-0.02	-0.03	0.56
I told close/trusted friends and/or family that I was going to be taking a psychedelic substance	0.24	0.17	0.29	0.03
I didn't do anything in particular to prepare for the psychedelic experience	0.39	0.01	0.02	0.33
I had prepared some strategies in case things started to get difficult during the psychedelic experience	0.16	0.06	-0.15	0.56

6.3 Items removed from PPS

Significant cross-loadings >0.20:

1. I was unaware of what to expect from the experience
2. I should have known more about the substance I was going to take
3. I was sure that keeping my intention in mind would help guide the psychedelic experience in a helpful direction
4. I went into the experience willing to learn more about the meaning behind my thoughts
5. I went into the experience with a curiosity about my own mind
6. I had significant doubts before going into the psychedelic experience
7. I felt afraid going into the psychedelic experience
8. I felt comfortable going into the psychedelic experience
9. I felt as though my mind and body would be 'strong enough' for the upcoming experience
10. I trusted the quality and purity of the of the substance
11. I felt ready to accept some big changes that might occur in myself as a result of the psychedelic experience
12. I was worried about becoming too different as a person after the psychedelic experience
13. I was afraid that I might change in a negative way after the psychedelic experience
14. I didn't do anything in particular to prepare for the psychedelic experience

Factor loadings < .30:

1. I had learned about the effects of the substance through conversations with other people
2. I expected the psychedelic experience to change my life
3. I was expecting the psychedelic experience to be easy and fun
4. I felt ready for the psychedelic experience
5. My friends and/or family were ready to support me through any changes I experienced
6. I dedicated time to preparing for the psychedelic experience
7. I told close/trusted friends and/or family that I was going to be taking a psychedelic substance

7.1 Reliability analysis of the hypothesized 4-factor model with sub-scale ω values if an item is removed and correlations of each item with the total of all other items in the subscale. Values in bold indicate items where overall reliability rises if the item is dropped. N=718.

Subscale reliability (McDonald's ω, 95%CI)	Item text	McDonald's ω if item dropped	Item-rest correlation
Knowledge-Expectation (ω=0.890, 95%CI=0.880-0.900)	I had done some of my own research into the effects of the psychedelic substance (e.g., reading articles/books, watching videos, listening to podcasts etc)	0.870	0.714
	I understood that the experience could evoke a range of intense emotions, from bliss to horror	0.867	0.740
	I understood that events from my past could surface into the psychedelic experience	0.879	0.676
	I knew that my experience would be somewhat unpredictable	0.864	0.755
	I was aware that the psychedelic experience might change me in some way	0.857	0.781
Intention-Preparation (ω=0.865, 95%CI=0.854-0.877)	I was ready to experience whatever 'came up' during the psychedelic experience	0.847	0.639
	I was prepared to deal with uncomfortable and challenging aspects of the psychedelic experience	0.909	0.735
	I felt ready to surrender to whatever the psychedelic experience would be	0.812	0.794
	I felt psychologically prepared for the psychedelic experience	0.825	0.752
	I was prepared for the physical effects of the psychedelic	0.818	0.768

	I trusted my own mind and body to safely process the experience	0.828	0.738
Psychophysical-Readiness (ω=0.828, 95%CI=0.813-0.844)	I had a clear intention for the psychedelic experience	0.799	0.617
	I had carefully contemplated my reasons for taking a psychedelic substance	0.812	0.588
	I spoke with a therapist/counsellor as part of my preparation for the psychedelic experience	0.765	0.709
	I engaged with specific preparation practices before the psychedelic experience (e.g., meditation, yoga, breathwork, journalling, diet, exercise)	0.758	0.704
Support-Planning (ω=0.799, 95%CI=0.782-0.817)	I felt a trusting, positive connection with the people who were going to be around me during the experience	0.787	0.477
	I felt the substance would be safe to take	0.794	0.467
	My family and/or friends were prepared and well-informed about the changes that could occur in me	0.734	0.647
	I had made a plan for what I would do in the hours and days after the psychedelic experience	0.736	0.647
	I had prepared some strategies in case things started to get difficult during the psychedelic experience	0.750	0.624

8.1 Group descriptives for high/low prep groups on the MEQ, CEQ, EBI, PTGI-SF, COES, and SWEBWBS outcomes

	Preparation Group	N	Mean	SD	t	p
**MEQ**	High	611	149.339	24.818	14.654	< .001
	Low	625	120.675	41.660		
**CEQ**	High	451	72.636	16.385	-8.647	< .001
	Low	523	83.874	23.024		
**EBI**	High	611	424.989	157.150	11.945	< .001
	Low	625	310.963	177.570		
**PTGI-SF**	High	611	39.858	7.916	14.437	< .001
	Low	625	31.174	12.639		
**COES**	High	611	55.043	9.517	17.235	< .001
	Low	625	42.213	15.813		
**SWEBWBS**	High	611	40.291	6.189	16.692	< .001
	Low	625	31.637	11.261		

9.1 Demographic characteristics of Retreat Survey participants

**Gender (n=46)**	Male	22 (47.83%)
	Female	24 (52.17%)
**Mean age in years (SD)**		44.02 (13.54)
**Lifetime psychedelic use**	Never before	25 (54.35%)
	On 1 occasion	9 (19.57%)
	On 2-5 occasions	6 (13.04%)
	On 6-10 occasions	2 (4.35%)
	On 11-20 occasions	3 (6.52%)
	On more than 20 occasions	1 (2.17%)
**Religion**	Not religious	25 (54.35%)
	Christian	8 (17.39%)
	Buddhism	3 (6.52%)
	Spiritual	10 (21.74%)
**Education**	Secondary/High School	2 (4.35%)
	Undergraduate	28 (60.87%)
	Postgraduate	16 (34.78%)
**Ethnicity**	White	32 (71.11%)
	Black	4 (8.89%)
	Asian	1 (2.22%)
	Latino/Hispanic	2 (4.44%)
	Mixed	6 (13.33%)

10.1 Bland-Altman plot: Intraindividual differences (n = 46) between mean PPS scores for test–retest, plotted against the average of the two scores. The central line represents the mean difference and the top and bottom lines display the 95% confidence interval.

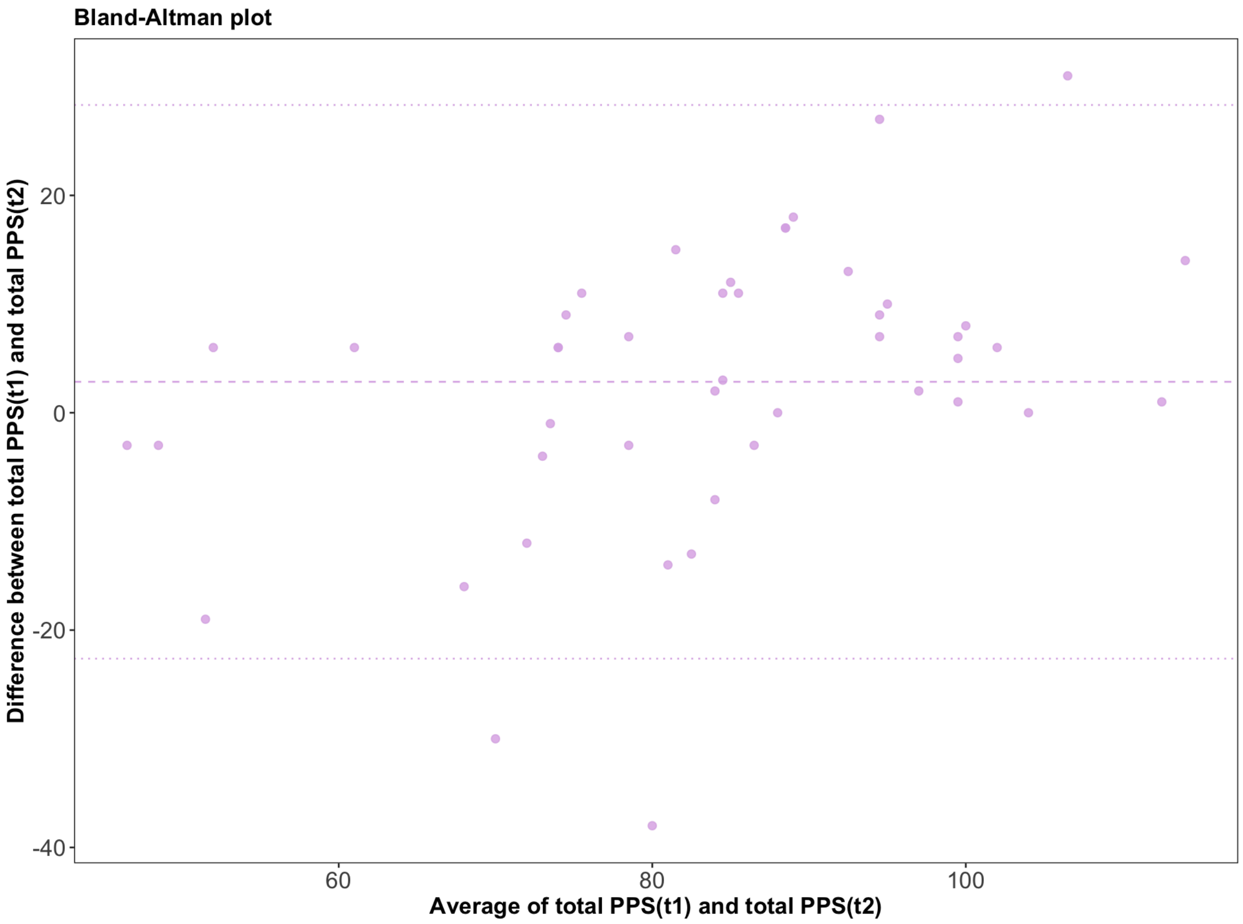

11.1 11D-ASC group descriptives

	Preparation Group	Mean	SD	t	p
**Global-ASC**	High	44.067	2.635	2.106	0.041
	Low	41.834	4.350		
**Experience of unity****	High	48.043	7.119	4.719	< .001
	Low	39.826	4.366		
**Spiritual experience****	High	44.043	4.876	3.545	< .001
	Low	38.130	6.341		
**Blissful state***	High	45.478	5.830	3.117	0.003
	Low	39.522	7.070		
**Insightfulness***	High	48.043	7.456	3.358	0.002
	Low	41.739	5.047		
**Disembodiment****	High	46.478	5.401	4.375	< .001
	Low	40.783	3.133		
**Impaired control and cognition**	High	49.217	12.087	0.234	0.816
	Low	48.261	15.404		
**Anxiety***	High	35.826	9.099	-2.348	0.023
	Low	43.609	13.034		
**Complex imagery**	High	41.870	10.015	1.205	0.235
	Low	37.957	11.933		
**Elementary imagery***	High	35.739	10.855	-3.432	0.001
	Low	45.783	8.893		
**Audio-visual synesthesia**	High	40.565	15.356	0.813	0.421
	Low	37.217	12.428		
**Changed meaning of percepts**	High	49.435	7.241	0.735	0.466
	Low	47.348	11.523		

** sig at $p<0.001$

* sig at $p<0.05$

11.2 11D-ASC Radar Plot

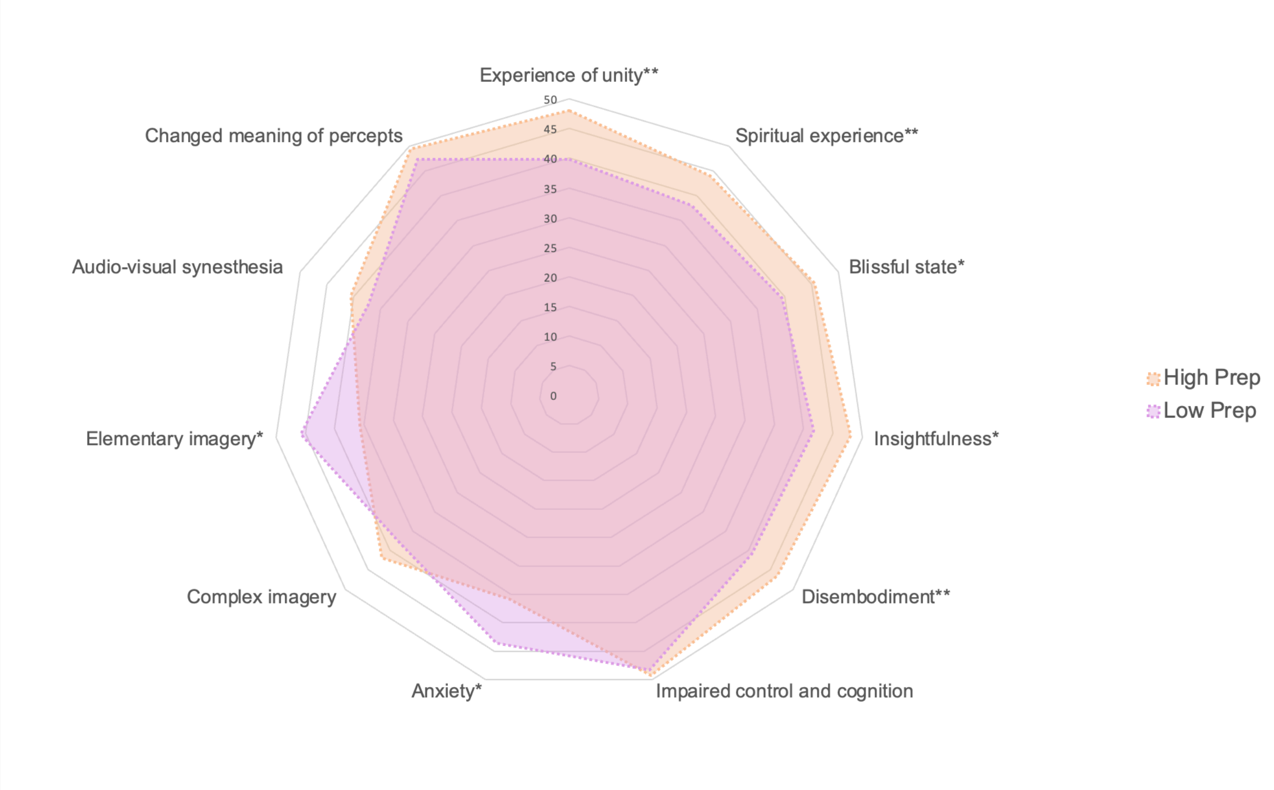

11.3 DASS group descriptives

	Group	Mean	SD	t	p
**Δ Depression**	High	-9.043	6.898	-3.526	< .001
	Low	-2.522	5.575		
**Δ Anxiety**	High	-9.565	6.535	-3.202	0.003
	Low	-2.826	7.691		
**Δ Stress**	High	-8.348	5.820	-3.162	0.003
	Low	-3.043	5.555		

Supplementary Material B
Title: Development and psychometric validation of a novel scale for measuring 'psychedelic preparedness'.
Authors: Rosalind G. McAlpine[1]*, George Blackburne[1,2], and Sunjeev K. Kamboj[1]

1.1 Psychedelic Preparedness Scale (PPS) (Prospective Version)

In relation to your upcoming psychedelic experience, indicate the degree to which you agree with the following statements.

1	2	3	4	5	6	7
Not at all	A little	More than a little	Moderately	Considerably	Very much	Completely

		Not at all	A little	More than a little	Moderately	Considerably	Very much	Completely
1	I have done some of my own research into the effects of the psychedelic substance (e.g., reading articles/books, watching videos, listening to podcasts etc)	1	2	3	4	5	6	7
2	I understand that the experience might evoke a range of intense emotions, from bliss to horror	1	2	3	4	5	6	7
3	I have a clear intention for the psychedelic experience	1	2	3	4	5	6	7
4	I have carefully contemplated my reasons for taking a psychedelic substance	1	2	3	4	5	6	7
5	I understand that events from my past could surface into the psychedelic experience	1	2	3	4	5	6	7
6	I know that my experience will be somewhat unpredictable	1	2	3	4	5	6	7
7	I have spoken with a therapist/counsellor as part of my preparation for the psychedelic experience	1	2	3	4	5	6	7
8	I am ready to experience whatever 'comes up' during the psychedelic experience	1	2	3	4	5	6	7
9	I am prepared to deal with uncomfortable and challenging aspects of the psychedelic experience	1	2	3	4	5	6	7

10	I feel ready to surrender to whatever the psychedelic experience will be	1	2	3	4	5	6	7
11	I feel psychologically prepared for the psychedelic experience	1	2	3	4	5	6	7
12	I am prepared for the physical effects of the psychedelic	1	2	3	4	5	6	7
13	I feel a trusting, positive connection with the people who are going to be around me during the experience	1	2	3	4	5	6	7
14	I feel the substance will be safe to take	1	2	3	4	5	6	7
15	I trust my own mind and body to safely process the experience	1	2	3	4	5	6	7
16	I am aware that the psychedelic experience might change me in some way	1	2	3	4	5	6	7
17	My family and/or friends are prepared and well-informed about the changes that could occur in me	1	2	3	4	5	6	7
18	I have engaged with specific preparation practices (e.g., meditation, yoga, breathwork, journaling, diet, exercise)	1	2	3	4	5	6	7
19	I have made a plan for what I will do in the hours and days after the psychedelic experience	1	2	3	4	5	6	7
20	I have prepared some strategies in case things started to get difficult during the psychedelic experience	1	2	3	4	5	6	7

**Factor**	**Item Numbers**
Knowledge-Expectation	1, 2, 5, 6, 16
Psychophysical-Readiness	8, 9, 10, 11, 12, 15
Intention-Preparation	3, 4, 7, 18
Support-Planning	13, 14, 17, 19, 20

1.2 Psychedelic Preparedness Scale (PPS) (Retrospective Version)

In relation to your psychedelic experience, indicate the degree to which you agree with the following statements.

1	2	3	4	5	6	7
Not at all	A little	More than a little	Moderately	Considerably	Very much	Completely

		Not at all	A little	More than a little	Moderately	Considerably	Very much	Completely
1	I had done some of my own research into the effects of the psychedelic substance (e.g., reading articles/books, watching videos, listening to podcasts etc)	1	2	3	4	5	6	7
2	I understood that the experience could evoke a range of intense emotions, from bliss to horror	1	2	3	4	5	6	7
3	I had a clear intention for the psychedelic experience	1	2	3	4	5	6	7
4	I had carefully contemplated my reasons for taking a psychedelic substance	1	2	3	4	5	6	7
5	I understood that events from my past could surface into the psychedelic experience	1	2	3	4	5	6	7
6	I knew that my experience would be somewhat unpredictable	1	2	3	4	5	6	7
7	I spoke with a therapist/counsellor as part of my preparation for the psychedelic experience	1	2	3	4	5	6	7
8	I was ready to experience whatever 'came up' during the psychedelic experience	1	2	3	4	5	6	7
9	I was prepared to deal with uncomfortable and challenging aspects of the psychedelic experience	1	2	3	4	5	6	7
10	I felt ready to surrender to whatever the psychedelic experience would be	1	2	3	4	5	6	7
11	I felt psychologically prepared for the psychedelic experience	1	2	3	4	5	6	7

12	I was prepared for the physical effects of the psychedelic	1	2	3	4	5	6	7
13	I felt a trusting, positive connection with the people who were going to be around me during the experience	1	2	3	4	5	6	7
14	I felt the substance would be safe to take	1	2	3	4	5	6	7
15	I trusted my own mind and body to safely process the experience	1	2	3	4	5	6	7
16	I was aware that the psychedelic experience might change me in some way	1	2	3	4	5	6	7
17	My family and/or friends were prepared and well-informed about the changes that could occur in me	1	2	3	4	5	6	7
18	I engaged with specific preparation practices before the psychedelic experience (e.g., meditation, yoga, breathwork, journaling, diet, exercise)	1	2	3	4	5	6	7
19	I had made a plan for what I would do in the hours and days after the psychedelic experience	1	2	3	4	5	6	7
20	I had prepared some strategies in case things started to get difficult during the psychedelic experience	1	2	3	4	5	6	7

**Factor**	**Item Numbers**
Knowledge-Expectation	1, 2, 5, 6, 16
Psychophysical-Readiness	8, 9, 10, 11, 12, 15
Intention-Preparation	3, 4, 7, 18
Support-Planning	13, 14, 17, 19, 20
